# Supplementary material for: IRX5 promotes DNA damage repair and activation of hair follicle stem cells
Source: Stem Cell Reports. 2023 Apr 20;18(5):1227–43. doi: 10.1016/j.stemcr.2023.03.013 (PMC10202659; doi:10.1016/j.stemcr.2023.03.013)
Supplement: Document S1. Figures S1–S7 and supplemental experimental procedures [file mmc1.pdf]

Stem Cell Reports, Volume *18*

Supplemental Information

IRX5 promotes DNA damage repair and activation of hair follicle stem cells

Jefferson K. Chen, Julie Wiedemann, Ly Nguyen, Zhongqi Lin, Mahum Tahir, Chi-Chung Hui, Maksim V. Plikus, and Bogi Andersen

Stem Cell Reports, Volume 18

Supplemental Information

IRX5 promotes DNA damage repair and activation of hair follicle stem cells

Jefferson K Chen, Julie Wiedemann, Ly Nguyen, Zhongqi Lin, Mahum Tahir, Chi-Chung Hui, Maksim V Plikus, Bogi Andersen.

Supplemental Figures

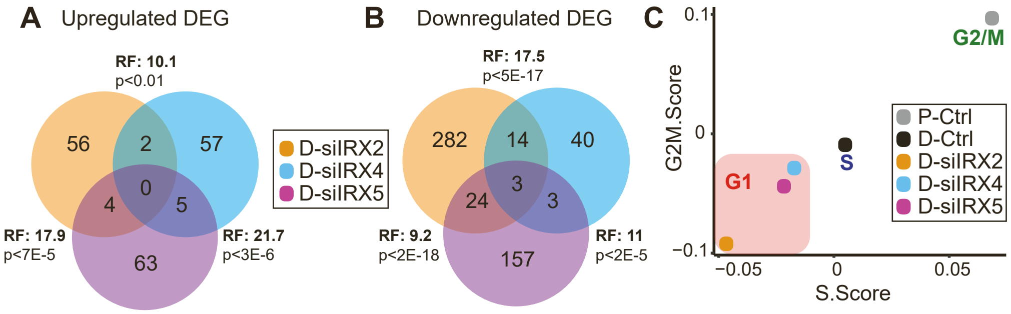

A
Upregulated DEG
RF: 10.1
p<0.01
56
2
57
0
4
5
63
RF: 17.9
p<7E-5
RF: 21.7
p<3E-6
D-siIRX2
D-siIRX4
D-siIRX5
B
Downregulated DEG
RF: 17.5
p<5E-17
282
14
40
3
24
3
157
RF: 9.2
p<2E-18
RF: 11
p<2E-5
C
0.1
0
−0.1
G2M.Score
−0.05
0
0.05
S.Score
G2/M
S
G1
P-Ctrl
D-Ctrl
D-siIRX2
D-siIRX4
D-siIRX5

Figure S1. IRX factors promote human keratinocyte proliferation, related to Figure 1. A-B) Venn diagrams showing overlap in DEG (upregulated, A; downregulated, B) after knockdowns with siIRX2, siIRX4, and siIRX5 in NHEK-D. **C**) Seurat scatter plot of cell cycle scores of control NHEK and NHEK-D after siRNA knockdowns. After knockdowns of IRX factors in NHEK-D, cell cycle gene expression becomes characteristic with the G1 stage of the cell cycle.

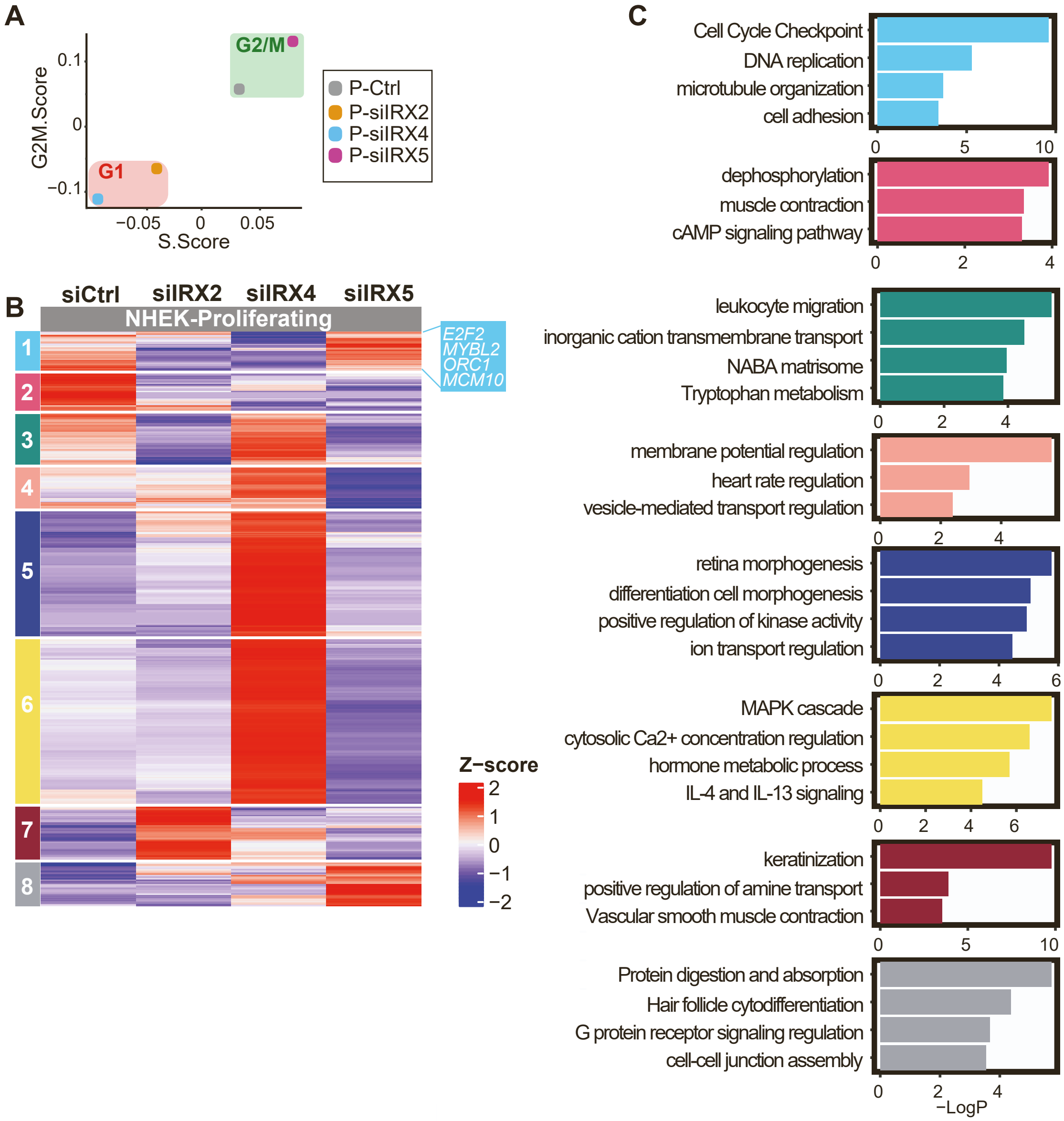

A
G2M.Score
S.Score
G2/M
G1
0.1
0
−0.1
−0.05
0
0.05
P-Ctrl
P-siIRX2
P-siIRX4
P-siIRX5
B
siCtrl
siIRX2
siIRX4
siIRX5
NHEK-Proliferating
1
2
3
4
5
6
7
8
E2F2
MYBL2
ORC1
MCM10
Z−score
2
1
0
−1
−2
C
Cell Cycle Checkpoint
DNA replication
microtubule organization
cell adhesion
dephosphorylation
muscle contraction
cAMP signaling pathway
leukocyte migration
inorganic cation transmembrane transport
NABA matrisome
Tryptophan metabolism
membrane potential regulation
heart rate regulation
vesicle-mediated transport regulation
retina morphogenesis
differentiation cell morphogenesis
positive regulation of kinase activity
ion transport regulation
MAPK cascade
cytosolic Ca2+ concentration regulation
hormone metabolic process
IL-4 and IL-13 signaling
keratinization
positive regulation of amine transport
Vascular smooth muscle contraction
Protein digestion and absorption
Hair follicle cytodifferentiation
G protein receptor signaling regulation
cell-cell junction assembly
−LogP

Figure S2. IRX factors promote human keratinocyte proliferation. A) Seurat scatter plot of cell cycle scores of control NHEK and NHEK-P after siRNA knockdowns. **B**) K-means clustering of the RNA-seq data in panel A. **C**) GO categories of each cluster in panel B.

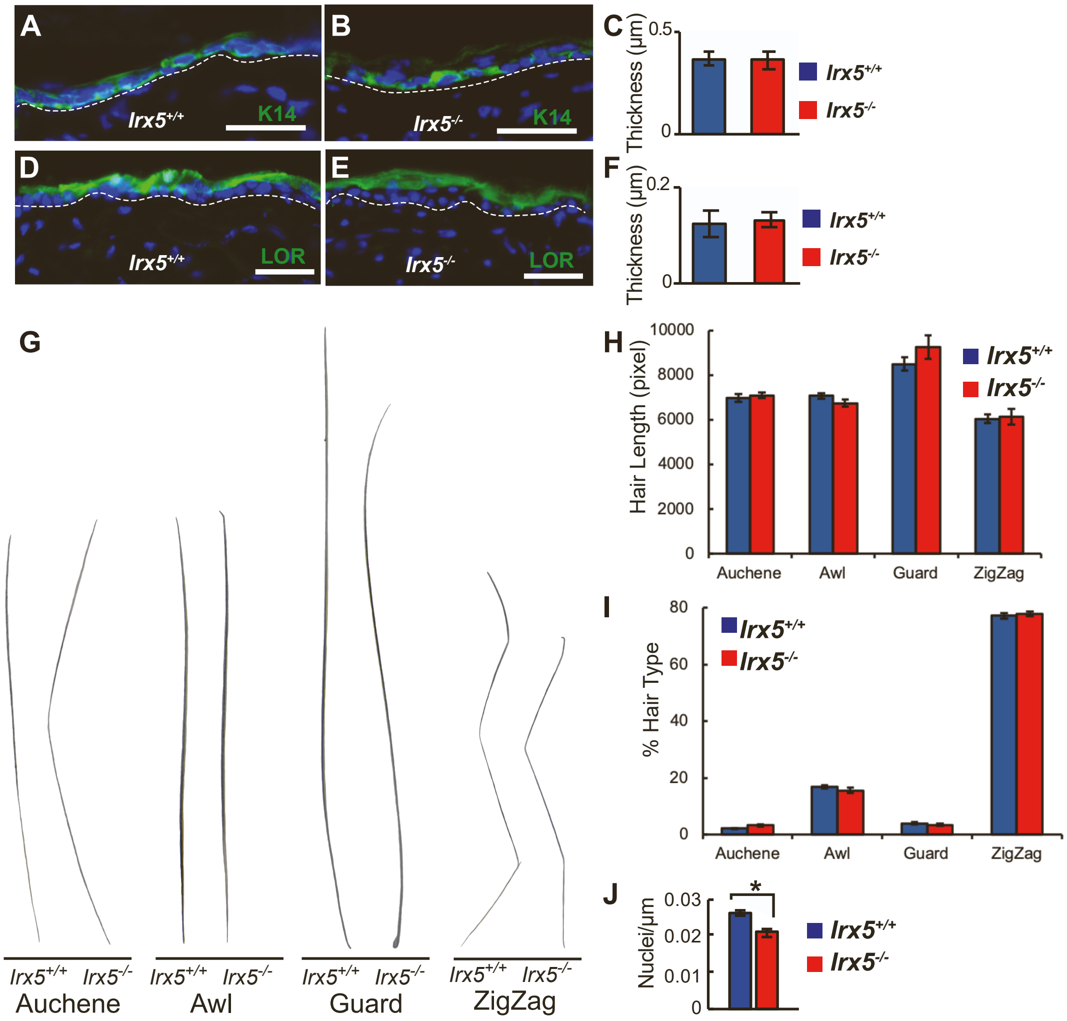
A
B
C
D
E
F
G
H
I
J
Irx5+/+
Irx5-/-
K14
LOR
Thickness (μm)
Hair Length (pixel)
% Hair Type
Nuclei/μm
Auchene
Awl
Guard
ZigZag
*

Figure S3. Mouse epidermis and distribution of hair fiber types is normal in *Irx5*$^{-/-}$ mice, related to Figure 2. A-B) Representative images of K14 immunofluorescent staining in P20 *Irx5*$^{+/+}$ and *Irx5*$^{-/-}$ littermates. **C**) Quantification of K14 staining thickness in *Irx5*$^{-/-}$ (n=2) and *Irx5*$^{+/+}$ P20 mice (n=2). **D-E**) Representative images of LOR immunofluorescent staining in P20 *Irx5*$^{+/+}$ and *Irx5*$^{-/-}$ littermates. **F**) Quantification of LOR staining thickness, in *Irx5*$^{-/-}$ (n=2) and *Irx5*$^{+/+}$ P20 mice (n=2). RF=representative factor. **G**) Representative samples of hair fibers plucked from P20 *Irx5*$^{+/+}$ and Irx5$^{-/-}$ littermates. 100 hair fibers each from *Irx5*$^{+/+}$ (n=2) and *Irx5*$^{-/-}$ (n=2) littermates were **H**) measured to identify differences in hair length and **I**) counted to identify differences in the proportion of hair fiber types. **J**) Quantification of nuclei per 1μm length in P20 *Irx5*$^{-/-}$ (n=3) and *Irx5*$^{+/+}$ mice (n=3); Two sample t-test at 95% CI; p=0.0147.

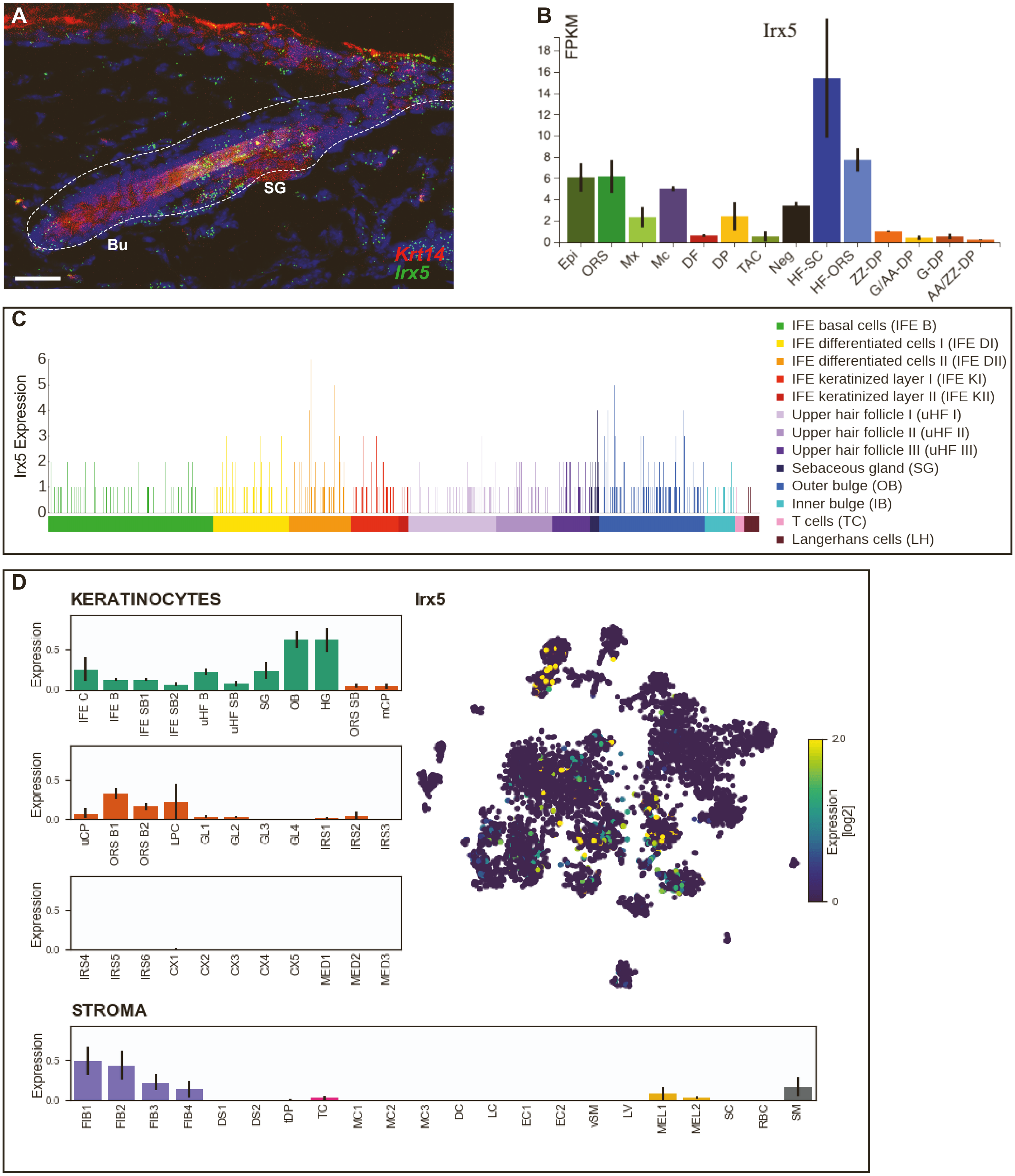

A
SG
Bu
Krt14
Irx5
B
Irx5
FPKM
Epi
ORS
Mx
Mc
DF
DP
TAC
Neg
HF-SC
HF-ORS
ZZ-DP
G/AA-DP
G-DP
AA/ZZ-DP
C
Irx5 Expression
IFE basal cells (IFE B)
IFE differentiated cells I (IFE DI)
IFE differentiated cells II (IFE DII)
IFE keratinized layer I (IFE KI)
IFE keratinized layer II (IFE KII)
Upper hair follicle I (uHF I)
Upper hair follicle II (uHF II)
Upper hair follicle III (uHF III)
Sebaceous gland (SG)
Outer bulge (OB)
Inner bulge (IB)
T cells (TC)
Langerhans cells (LH)
D
KERATINOCYTES
Irx5
Expression
IFE C
IFE B
IFE SB1
IFE SB2
uHF B
uHF SB
SG
OB
HG
ORS SB
mCP
uCP
ORS B1
ORS B2
LPC
GL1
GL2
GL3
GL4
IRS1
IRS2
IRS3
IRS4
IRS5
IRS6
CX1
CX2
CX3
CX4
CX5
MED1
MED2
MED3
STROMA
FIB1
FIB2
FIB3
FIB4
DS1
DS2
tDP
TC
MC1
MC2
MC3
DC
LC
EC1
EC2
vSM
LV
MEL1
MEL2
SC
RBC
SM
Expression [log2]

Figure S4. IRX5 is expressed in bulge cells, related to Figure 2. A) Representative sample of *Krt14* and *Irx5* RNA-FISH in normal P20 mouse hair follicle. Scale bar=20μm. Irx5 gene expression in **B**) sorted cells of P5 postnatal mouse skin from Rezza et al. (2016), **C**) second telogen mouse skin from Joost et al (2016), and **D**) mouse skin from Joost et al (2020).

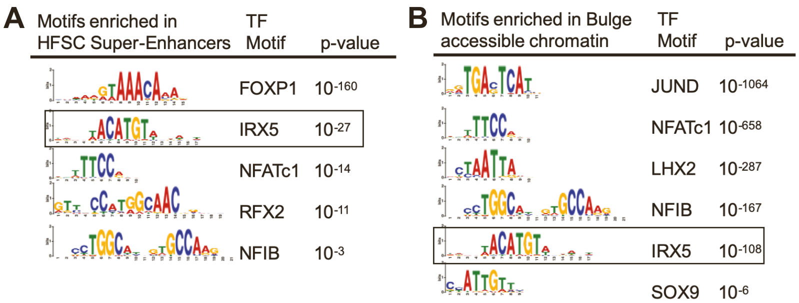
A
Motifs enriched in HFSC Super-Enhancers
TF Motif
p-value
FOXP1 10^{-160}
IRX5 10^{-27}
NFATc1 10^{-14}
RFX2 10^{-11}
NFIB 10^{-3}
B
Motifs enriched in Bulge accessible chromatin
TF Motif
p-value
JUND 10^{-1064}
NFATc1 10^{-658}
LHX2 10^{-287}
NFIB 10^{-167}
IRX5 10^{-108}
SOX9 10^{-6}

Figure S5. IRX5 motifs are enriched in HFSC gene-regulatory chromatin regions. Enriched motifs previously reported and IRX5 motif were reanalyzed using data from **A**) Adam et al. (2015) identified bulge super-enhancers and **B**) Adam et al. (2018) isolated bulge ATAC-seq.

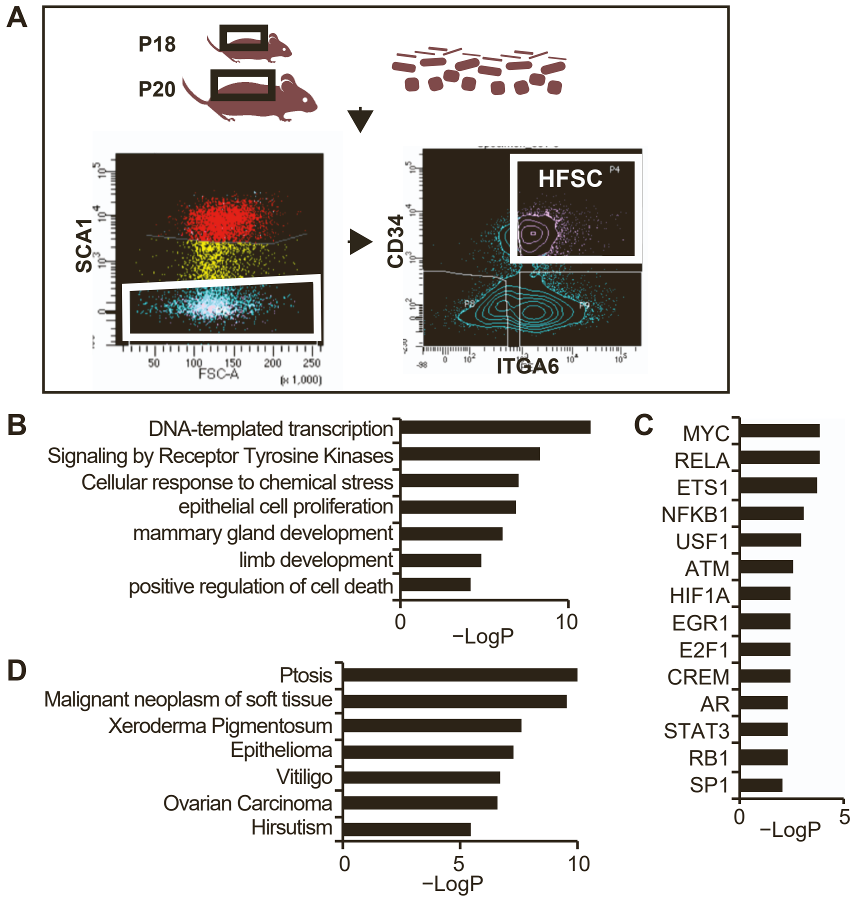
A
P18
P20
SCA1
FSC-A
50
100
150
200
250
(x 1,000)
CD34
HFSC
ITGA6
B
DNA-templated transcription
Signaling by Receptor Tyrosine Kinases
Cellular response to chemical stress
epithelial cell proliferation
mammary gland development
limb development
positive regulation of cell death
0
10
−LogP
C
MYC
RELA
ETS1
NFKB1
USF1
ATM
HIF1A
EGR1
E2F1
CREM
AR
STAT3
RB1
SP1
0
5
−LogP
D
Ptosis
Malignant neoplasm of soft tissue
Xeroderma Pigmentosum
Epithelioma
Vitiligo
Ovarian Carcinoma
Hirsutism
0
5
10
−LogP

Figure S6. Hair Follicle Stem Cell isolation, related to Figure 3. A) P18 and P20 *Irx5*$^{+/+}$ and *Irx5*$^{-/-}$ mice back skin was processed into a single cell suspension and FACS sorted for Sca1- CD34+ ITGA6+ HFSC. RNA sequencing was conducted with the isolated cells. *Irx5*$^{-/-}$ DEG which remained consistent between P18 and P20 (Cluster 3, 6, and 8 from Figure 3C) were analyzed together for **B**) GO, **C**) predicted transcriptional regulators, and **D**) mouse phenotype ontology.

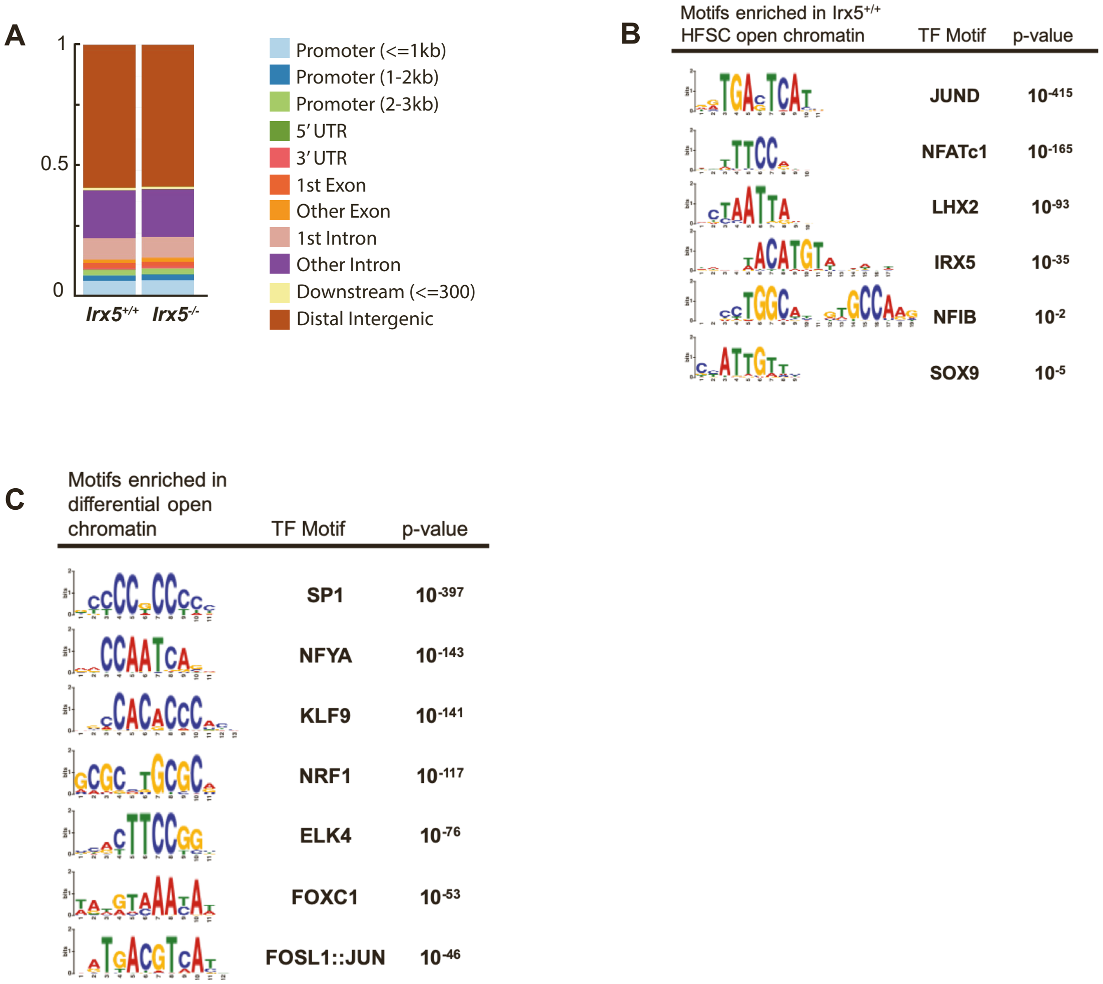

A
1
0.5
0
Irx5+/+
Irx5-/-
Promoter (<=1kb)
Promoter (1-2kb)
Promoter (2-3kb)
5′ UTR
3′ UTR
1st Exon
Other Exon
1st Intron
Other Intron
Downstream (<=300)
Distal Intergenic
B
Motifs enriched in Irx5+/+ HFSC open chromatin
TF Motif
p-value
JUND 10-415
NFATc1 10-165
LHX2 10-93
IRX5 10-35
NFIB 10-2
SOX9 10-5
C
Motifs enriched in differential open chromatin
TF Motif
p-value
SP1 10-397
NFYA 10-143
KLF9 10-141
NRF1 10-117
ELK4 10-76
FOXC1 10-53
FOSL1::JUN 10-46

Figure S7. P20 *Irx5*$^{-/-}$ HFSC differential open chromatin is enriched in NFY-A and SP1 binding sites, related to Figure 5. A) Genomic distribution of all open chromatin regions identified in P20 *Irx5*$^{-/-}$ HFSC and P20 *Irx5*$^{+/+}$ HFSC. **B**) Enriched motifs identified in P20 *Irx5*$^{+/+}$ HFSC. **C**) Enriched motifs identified in differential open chromatin of P20 *Irx5*$^{-/-}$ HFSC.

Supplemental Experimental Procedures

Mice

Irx5$^{-/-}$ mice and genotyping were described previously (Gaborit et al., 2012). Mice were maintained on standard 12h light cycles with ad libitum food and water. Animal experiments were performed in accordance to University of California, Irvine Institutional Animal Care and Use Committee (Protocol No. AUP-19-012). All mouse experiments were conducted on sex-matched littermates. EdU proliferation analysis was conducted with Click-iT EdU Cell Proliferation Kit (C10337). 0.5mg/mL of EdU was administered IP and mice were euthanized 2h later.

To observe anagen hair follicle growth, the back fur was shaved at P20 and mice were anesthetized and imaged every 3d. Hair follicle rescue experiments were conducted with AZD4547 (Selleckchem S2801) prepared in 4% DMSO, 5% PEG300, and 5% Tween80. AZD4547 (6mg/kg) was administered IP every 3d. Statistical analysis of back fur coverage was conducted with two-sided Kolmogorov-Smirnov test.

Tissue and Cell Isolation

P18 and P20 back skin was dissected and incubated in 0.25% trypsin (Invitrogen 1505-065) for 2h at 34C. The epidermal side was mechanically separated and resuspended in 2% FBS PBS. Debris was removed with 40uM and 70uM mesh filters. Whole epidermis samples were processed for bulk RNA-seq. For HFSC bulk RNAseq, cells were labeled with CD49f (eBioscience 12-0495), Ly-6A/E (eBioscience 45-5981-82), CD34 (eBioscience 50-0341-82), and DAPI. Ly-6A/E- CD34+ CD49f+ HFSC were isolated for bulk RNAseq and ATACseq.

Cell Culture

NHEK were obtained from donor neonatal foreskin and grown in Keratinocyte Serum Free Media supplemented with Epidermal Growth Factor and Bovine Pituitary Extract (Life Technologies). Individual Dharmacon on-TARGETplus siRNAs [negative control

(4390843), IRX2 (s45799, s45801), IRX3 (s35710, s35712), IRX4 (s55542, s27096), IRX5 (s20056, s20054)] were validated and siRNAs with at least 70% knockdown efficiency were pooled. 30nM pooled siRNAs were transfected into semi-confluent monolayers with Lipofectamine RNAi Max (Life Technologies) in OptiMEM medium. Twelve hours after transfection, 1.8mM Ca2+ was added to the medium to induce differentiation. RNA lysate was collected 72h after transfection.

Bulk RNA-sequencing

RNA quality was determined with Aglient Bioanalyzer; samples with RNA Integrity Number > 8 were used. Library preparation with Illumina TrueSeq library preparation kit and single end Illumina HiSeq 2500 sequencing was performed. Read alignment was preformed using Kallisto/0.46.2, and Deseq2 was used to identify DEG. GO was conducted using Metascape (Zhou et al., 2019).

Quantitative real time PCR was used for validation of RNA-sequencing data. cDNA was prepared with iScript cDNA kit and RT-PCR was performed with Ssofast EvaGreen (Biorad) reagent in CX384 Real-Time PCR system (Biorad). GAPDH was used to normalize gene expression between samples.

ATAC-seq

HFSC (100,000 cells/replicate) were FACS sorted and lysed according to published protocols (Buenrostro et al., 2015). Nuclei were incubated in 50uL of Tn5 transposition buffer for 30min at 37C. DNA isolation was performed with Qiagen MinElute Cleanup kit. Library preparation with Illumina TrueSeq library preparation kit and paired end Illumina HiSeq 2500 sequencing (80million reads per sample) was performed at the University of California, Irvine High Throughput Genomics Facility. FASTQC was used to check read quality. Adapters were removed with cutadapt v2.9 (parameter: -a CTGTCTCTTATACACATCT -A CTGTCTCTTATACACATCT) and sequences were aligned and mapped to mm10 (UCSC) with Bowtie2 (Langmead and Salzberg, 2012). Reads mapped to mitochondrial DNA were excluded. PCR duplicates were removed based on the list of duplicate generated from Genrich (v0.6, available at

github.com/jsh58/Genrich). Properly paired and mapped reads with mapping quality over 30 were kept for downstream analysis. To explore the feature of the uniquely mapped reads, deeptools (v3.4.2, parameter: -of bigwig --effectiveGenomeSize 2652783500 --normalizeUsing RPKM -e --ignoreDuplicates -bs 10) was used to convert aligned reads into a coverage track in BigWig format after read normalization based on RPKM. ChIPseeker (v1.24.0) was used to annotate the function of peaks and genomic region ontology was conducted with Genomic Regions Enrichment of Annotations Tool (GREAT) (McLean et al., 2010). ATACseq and RNAseq co-analysis was conducted with BETA using standard BETA plus parameters (Wang et al., 2013). Motif analysis was conducted with MEME Suit 5.5.0 using predicted IRX5 motif (JASPAR 2020 #PH0086.1).

RNA and protein detection

For immunofluorescence localization of protein, fresh tissue samples were harvested and embedded in OCT. 10uM sections were fixed in acetone at -20C for 10min, fixed in 4% PFA for 10min, permeabilized in 0.3% TritonX-100 for 10min, and blocked in 0.5% BSA PBS for 1h. Treated tissue was then incubated in primary antibody Krt14 (Abcam ab7800), Krt10 (Covance PRB-159P), and H2AX (cell signaling 9718) overnight at 4C. Secondary antibodies were incubated at room temperature for 1h. Images were captured with Keyence BZ-X700 or Nikon LSM780 confocal microscope.

For RNA FISH, fresh frozen 10uM thick OCT sections were processed and stained using RNAscope Multiplex Fluorescent Detection Kit v1 according to manufacturer's instructions. Processed samples were counterstained and preserved with ProLong Gold antifade reagent with DAPI. Images were captured on Nikon LSM780 confocal microscope.

Comparable images were post-processed in batches using the same maximum intensity projection and brightness setting for consistency with Zeiss Zen blue. Two to six biological replicates were analyzed for each marker, with each biological replicate analyzed from five to ten hair follicles. Nuclei density within the epidermis was quantified by dividing the total nuclei count by the length of the epidermis. Immunofluorescent

intensity was determined based on the RGB levels for each respective marker in the defined ROI relative to the intensity of the DAPI intensity. Each ROI was defined through the ImageJ Polygon Tool. P values were calculated using comparison of means at 95% confidence interval.

Supplemental References

Gaborit, N., Sakuma, R., Wylie, J.N., Kim, K.-H., Zhang, S.-S., Hui, C.-C., and Bruneau, B.G. (2012). Cooperative and antagonistic roles for Irx3 and Irx5 in cardiac morphogenesis and postnatal physiology. Development *139*, 4007-4019. 10.1242/dev.081703.

Langmead, B., and Salzberg, S.L. (2012). Fast gapped-read alignment with Bowtie 2. Nature Methods *9*, 357-359. 10.1038/nmeth.1923.

McLean, C.Y., Bristor, D., Hiller, M., Clarke, S.L., Schaar, B.T., Lowe, C.B., Wenger, A.M., and Bejerano, G. (2010). GREAT improves functional interpretation of cis-regulatory regions. Nature Biotechnology *28*, 495-501. 10.1038/nbt.1630.

Wang, S., Sun, H., Ma, J., Zang, C., Wang, C., Wang, J., Tang, Q., Meyer, C.A., Zhang, Y., and Liu, X.S. (2013). Target analysis by integration of transcriptome and ChIP-seq data with BETA. Nature Protocols *8*, 2502-2515. 10.1038/nprot.2013.150.

Zhou, Y., Zhou, B., Pache, L., Chang, M., Khodabakhshi, A.H., Tanaseichuk, O., Benner, C., and Chanda, S.K. (2019). Metascape provides a biologist-oriented resource for the analysis of systems-level datasets. Nature Communications *10*, 1523. 10.1038/s41467-019-09234-6.
