# Supplementary material for: IRX5 promotes DNA damage repair and activation of hair follicle stem cells
Source: Stem Cell Reports. 2023 Apr 20;18(5):1227–43. doi: 10.1016/j.stemcr.2023.03.013 (PMC10202659; doi:10.1016/j.stemcr.2023.03.013)
Supplement: Document S3. Article plus supplemental information [file mmc3.pdf]

Stem Cell Reports
Article
OPEN ACCESS

IRX5 promotes DNA damage repair and activation of hair follicle stem cells

Jefferson K. Chen,[1] Julie Wiedemann,[1,2] Ly Nguyen,[1] Zhongqi Lin,[1] Mahum Tahir,[1] Chi-Chung Hui,[3] Maksim V. Plikus,[4] and Bogi Andersen[1,*]
[1]Departments of Biological Chemistry and Medicine, Division of Endocrinology, School of Medicine, University of California, Irvine, Irvine, CA, USA
[2]Mathematical, Computational and Systems Biology (MCSB) Program, University of California, Irvine, Irvine, CA, USA
[3]Program in Developmental & Stem Cell Biology, The Hospital for Sick Children and Department of Molecular Genetics, University of Toronto, Toronto, ON, Canada
[4]Department of Developmental and Cell Biology, University of California, Irvine, Irvine, CA 92697, USA
*Correspondence: bogi@uci.edu
https://doi.org/10.1016/j.stemcr.2023.03.013

SUMMARY

The molecular mechanisms allowing hair follicles to periodically activate their stem cells (HFSCs) are incompletely characterized. Here, we identify the transcription factor IRX5 as a promoter of HFSC activation. *Irx5*$^{-/-}$ mice have delayed anagen onset, with increased DNA damage and diminished HFSC proliferation. Open chromatin regions form near cell cycle progression and DNA damage repair genes in *Irx5*$^{-/-}$ HFSCs. DNA damage repair factor BRCA1 is an IRX5 downstream target. Inhibition of FGF kinase signaling partially rescues the anagen delay in *Irx5*$^{-/-}$ mice, suggesting that the *Irx5*$^{-/-}$ HFSC quiescent phenotype is partly due to failure to suppress *Fgf18* expression. Interfollicular epidermal stem cells also show decreased proliferation and increased DNA damage in *Irx5*$^{-/-}$mice. Consistent with a role for IRX5 as a promoter of DNA damage repair, we find that IRX genes are upregulated in many cancer types and that there is a correlation between *IRX5* and *BRCA1* expression in breast cancer.

INTRODUCTION

First discovered in *Drosophila*, Iroquois (IRX) homeodomain transcription factors function early in development to differentiate the dorsal head and mesothorax but also remain active in later stages of development to subdivide these territories (Gómez-Skarmeta and Modolell, 1996). Extensive studies have characterized the six vertebrate IRX factors as developmental regulators, with key roles in myocardial development (Bruneau et al., 2001), bone formation (Cain et al., 2016), and neuronal development (Briscoe et al., 2000). In humans, the FTO locus, which is associated with obesity, regulates the expression of *IRX3* and *IRX5* in preadipocytes and brain regions (Sobreira et al., 2021; Son et al., 2021). There are no reports of IRX's role in epidermal keratinocytes and hair follicle development.

A few studies, primarily in cancer, have characterized the role of IRX5 in the cell cycle, apoptosis, and cell migration. IRX5 is a tumorigenic factor in hepatocellular carcinoma (Zhu et al., 2020), tongue squamous cell carcinoma (Huang et al., 2018), and non-small cell lung cancer (Zhang et al., 2018). Little is known about the role of IRX5 in cell proliferation during homeostatic tissue maintenance and the mechanisms by which IRX5 controls cell proliferation. The epidermis and hair follicles rely on active stem cell proliferation for tissue homeostasis, making the skin a suitable organ to investigate how IRX5 controls cell proliferation.

Proliferating cells are prone to replication errors. Hence, DNA damage repair is critical in long-lived stem cells that maintain tissues through high turnover of cells. Hair follicle stem cells (HFSCs) and interfollicular epidermal stem cells (EpiSCs) also accumulate mutations from environmental insults, including ultraviolet radiation. Unsurprisingly, HFSCs and EpiSCs have evolved robust DNA damage repair mechanisms that make them resistant to DNA damage (Sotiropoulou et al., 2010, 2013). Little is known about the transcriptional and epigenetic regulators that confer DNA damage resistance to HFSCs and EpiSCs.

One factor implicated in DNA damage repair in HFSCs is BRCA1, a tumor suppressor with roles in double-stranded DNA break resection, DNA repair, chromatin remodeling, and cell cycle checkpoint regulation (Sotiropoulou et al., 2013; Wu et al., 2010; Zhu et al., 2011). In the absence of BRCA1, HFSCs are depleted through DNA damage-mediated apoptosis, rendering mice hairless (Sotiropoulou et al., 2013). The regulators of *BRCA1* in hair follicle growth are unknown.

Here, we show that IRX5 is a pro-proliferative factor necessary for DNA damage repair and HFSC activation. We identify *Brca1* and other DNA repair genes as targets of IRX5, consistent with the hair follicle phenotype of *Brca1*$^{-/-}$ mice (Sotiropoulou et al., 2013). We also identify upregulation of hair cycle inhibitor FGF18 as a mechanism that partially mediates quiescence due to DNA damage in *Irx5*$^{-/-}$ HFSCs.

RESULTS

Knockdown of IRX downregulates cell cycle genes in human keratinocytes

Investigating the differentiation of normal human epidermal keratinocytes (NHEKs), we identified IRX

This is an open access article under the CC BY license (http://creativecommons.org/licenses/by/4.0/).

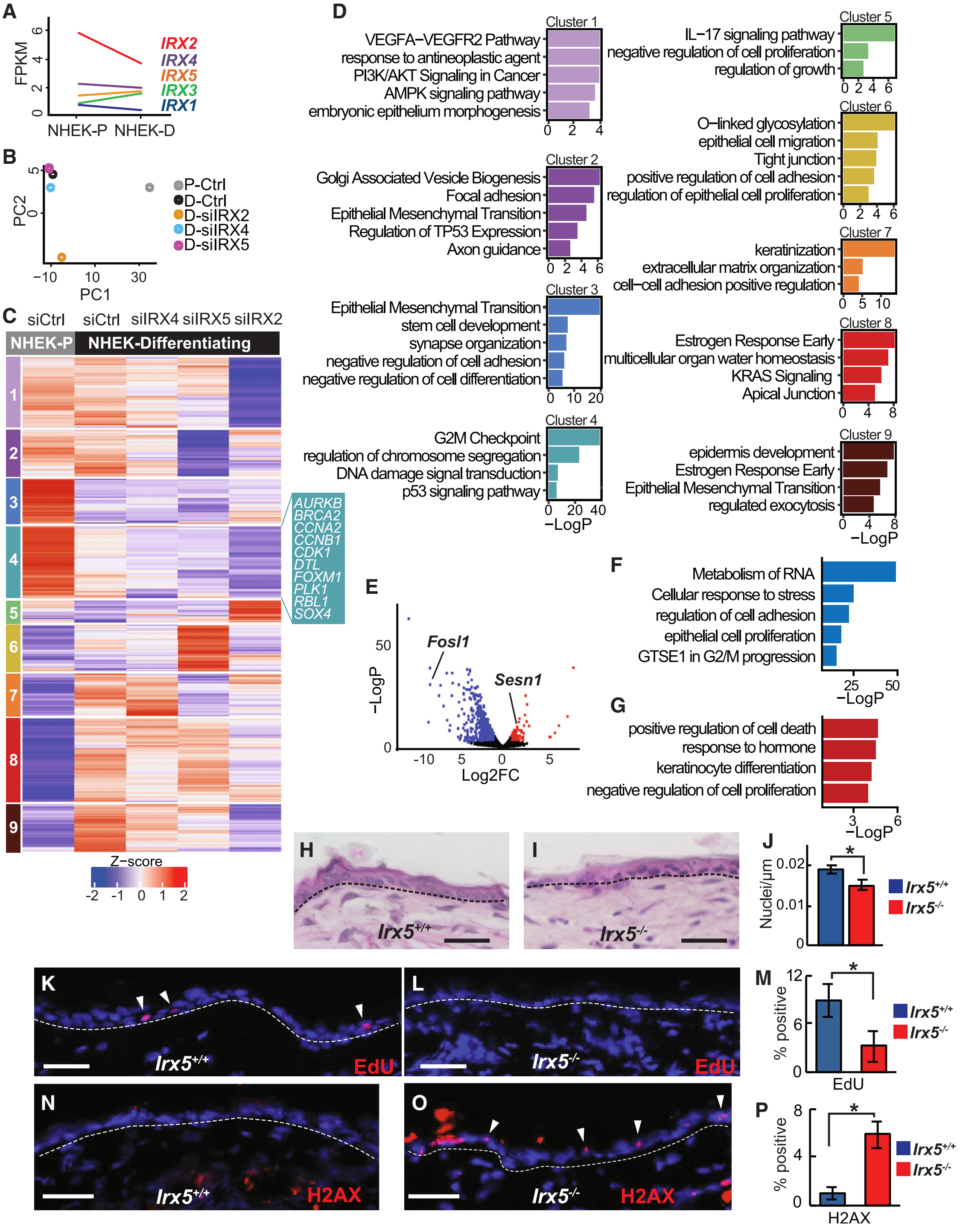

Figure 1. IRX promotes keratinocyte proliferation and DNA damage repair

(A) IRX1-5 gene expression in proliferating (NHEK-P) and differentiated (NHEK-D) cultured keratinocytes.
(B) PCA of RNA-seq gene expression data after differentiation and siIRX knockdown in differentiating keratinocytes.
(C) K-means clustering of the RNA-seq data in (B).
(D) GO categories of each cluster in (C).
(E) Volcano plot of DEGs, comparing P20 epidermis in *Irx5*$^{-/-}$ mice (n = 2) and *Irx5*$^{+/+}$ mice (n = 2).
(F) GO of downregulated genes in (E).

(legend continued on next page)

binding sites in the gene regulatory regions of the pro-differentiation Grainyhead like transcription factor 3 (GRHL3), suggesting that IRX could regulate epidermal differentiation (Klein et al., 2017). To characterize IRX's role in epidermal differentiation, we used calcium-induced differentiation of NHEKs (Hopkin et al., 2012). *IRX1–IRX5* are expressed in proliferating and differentiating NHEKs. *IRX4*, *IRX2*, and *IRX5* are most highly expressed, and *IRX2* is downregulated upon differentiation (Figure 1A).

We then used siRNA knockdown and RNA sequencing (RNA-seq) to characterize the IRX2-, IRX4-, and IRX5-regulated genes in NHEKs. Principal component analysis (PCA) of these data (Figure 1B) shows that the greatest difference is between proliferating (NHEK-Ps) and differentiating (NHEK-Ds) NHEKs, as revealed by the variance in the PC1 axis. Knockdown of IRX factors in NHEK-Ds had less effect as revealed by the variance in the PC2 axis.

Comparing NHEK-Ds to NHEK-Ps, we identified 289 differentially expressed genes (DEGs) (242 upregulated, 47 downregulated; Data S1). After knockdown of IRX2, IRX4, and IRX5 in NHEK-Ds, we identified, respectively, 385 (62 upregulated, 323 downregulated; Data S2), 124 (64 upregulated, 60 downregulated; Data S3), and 259 (72 upregulated, 187 downregulated; Data S4) DEGs (Figures S1A and S1B). Knockdown of the three different IRX factors largely affect distinct genes (Figure S1B).

In the combined data, K-means clustering identified nine distinct expression groups containing a total of 821 genes (Figures 1C and 1D, Data S5). Cluster 1 and 2 genes have similar expression in NHEK-Ps and NHEK-Ds, whereas cluster 3–5 and cluster 6–9 genes are down- and upregulated, respectively, in NHEK-Ds compared with NHEK-Ps (Figures 1C and 1D). Cluster 4 genes, which are downregulated upon differentiation, are enriched for gene ontology (GO) categories G2M checkpoint, regulation of chromosome segregation, DNA damage signal transduction, and p53 signaling pathway. These genes are downregulated after knockdown of individual IRXs, suggesting that IRX factors promote keratinocyte cell division and DNA repair. These genes include pro-proliferation genes *RBL1*, *CDC6*, *MYBL2*, *CDK1*, *CDK6*, *CCNA1*, *CCNA2*, *CCNB1*, and *WSB2*, as well as DNA damage repair genes *BRCA2* and *FOXM1* (Figures 1C and 1D). Knockdown of IRX2, IRX4, or IRX5 all led to increased G1 phase gene expression (Figure S1C) based on Seurat's (Stuart et al., 2019) cell cycle scoring function.

Knockdown of IRX2 and IRX4 in NHEK-Ps resulted in G1 phase gene expression (Figure S2A). In contrast to the effect in NHEK-Ds, knockdown of IRX5 did not promote the G1 state in NHEK-Ps, suggesting that its effects on *in vitro* keratinocyte proliferation are more prominent at the beginning of differentiation. We identified 776 DEGs ($p < 0.05$), which clustered into eight unique expression patterns among proliferating control and proliferating siIRX knockdown (Figure S2B), including G1/S and G2/M transition genes that are downregulated upon IRX knockdown (Figure S2C). Collectively, these data suggest that IRX factors regulate genes in human keratinocytes that promote cell cycle progression.

Irx5$^{-/-}$ basal epidermal keratinocytes have decreased proliferation and increased DNA damage

As *Irx2*, *Irx3*, and *Irx5* are the highest expressing *Irx* in mouse epidermis (Data S6), we turned to *Irx5*$^{-/-}$ mice (Cheng et al., 2005). We dissociated epidermis from postnatal (P) day 20 *Irx5*$^{-/-}$ (n = 2) and *Irx5*$^{+/+}$ (n = 2) mice into single cell suspension. Through RNA-seq, we identified 478 upregulated and 1,513 downregulated genes in the *Irx5*$^{-/-}$ epidermis (Figure 1E, and Data S6). As with the *in vitro* NHEK studies, GO term analysis of downregulated genes revealed significant enrichment in pro-proliferation categories such as RNA metabolism, translation, and cell cycle (Figure 1F). Cell proliferation genes included *Pcna*, *Cdk11b*, *Cdkn1a*, *Jak2*, *Akt2*, *Fosl1*, *Sox9*, *Fgfbp1*, *Bmp2*, *Notch1*, *Tgm1*, *Vegfa*, *Jun*, and *Myc*. Upregulated genes in *Irx5*$^{-/-}$ epidermis contained GO categories such as keratinocyte differentiation, negative regulation of cell proliferation, hair follicle development, and positive regulation of cell death (Figure 1G).

The structure of the P20 *Irx5*$^{-/-}$ back epidermis is normal (Figures 1H and 1I), with slightly decreased basal cell density ($p = 0.0225$) (Figure 1J). Consistent with the gene expression changes, we found decreased epidermal proliferation in the *Irx5*$^{-/-}$ mice. At P20, the *Irx5*$^{+/+}$ (n = 4) and the *Irx5*$^{-/-}$ (n = 4) epidermis contained, respectively, 8% and 2% EdU^{+} cells ($p = 0.0002$) (Figures 1K-1M). Also consistent with the *in vitro* and *in vivo* gene expression data, we detected increased γH2AX staining in the *Irx5*$^{-/-}$ epidermis (Figures 1N and 1O); γH2AX marks unrepaired

(G) GO of upregulated genes in (E).
(H and I) Representative H&E images of P20 *Irx5*$^{+/+}$ and *Irx5*$^{-/-}$ littermates.
(J) Quantification of nuclei per 1-μm length in *Irx5*$^{-/-}$ (n = 5) and *Irx5*$^{+/+}$ P20 mice (n = 6). Two-sample t test at 95% CI; p = 0.0225.
(K and L) Representative images of EdU staining in P20 littermates. Scale bar represents 25 μm.
(M) Quantification of EdU^{+} cells in the epidermis of *Irx5*$^{-/-}$ (n = 4) and *Irx5*$^{+/+}$ mice (n = 4). Two-sample t test at 95% CI; p = 0.0002.
(N and O) Representative images of H2AX immunofluorescent staining in P20 littermates. Scale bar represents 25 μm.
(P) Quantification of H2AX^{+} cells in keratinocytes of *Irx5*$^{-/-}$ (n = 3) and *Irx5*$^{+/+}$ mice (n = 3). Two-sample t test at 95% CI; p = 0.0001.

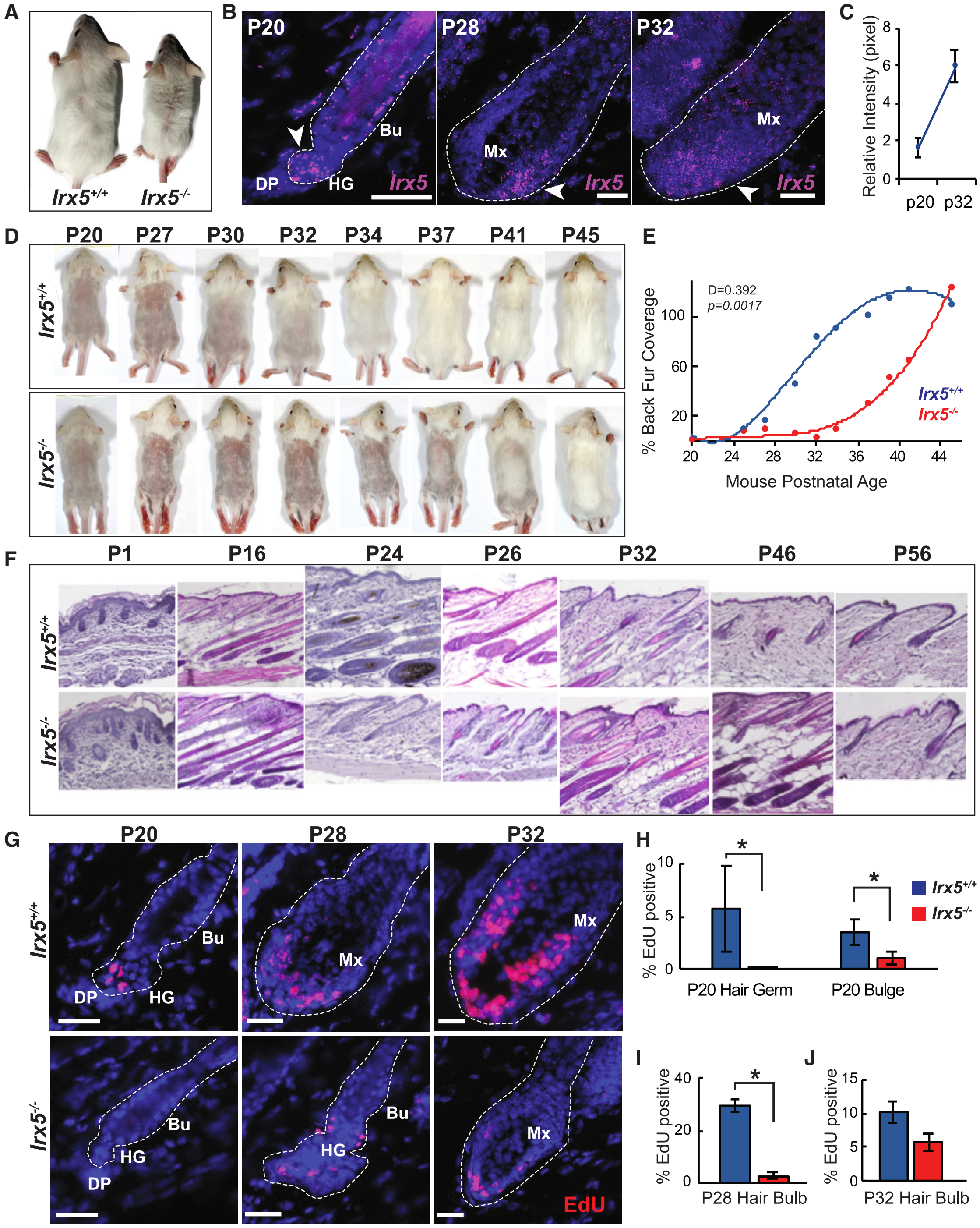

Figure 2. IRX5 promotes anagen initiation in mice

(A) P55 *Irx5*$^{+/+}$ and *Irx5*$^{-/-}$ littermates.

(B) *Irx5* RNA-FISH in normal mouse hair follicles from P20, P28, and P32. Arrows indicate areas of strong Irx5 expression. Scale bar represents 25 μm.

(C) Quantification of Irx5 mRNA expression intensity in the hair follicle lower bulge at P20 (n = 4) and P32 (n = 2).

(D) Hair regrowth after shaving. Shown are representative *Irx5*$^{-/-}$ and *Irx5*$^{+/+}$ littermates.

(E) Quantification of dorsal hair growth in three sets of littermates (n = 6 per genotype). Two-sided Kolmogorov-Smirnov test; D = 0.392, p = 0.001724.

(legend continued on next page)

double-stranded breaks. There was no γH2AX staining in the *Irx5*$^{+/+}$ epidermis (n = 3), whereas 5% of keratinocytes in the *Irx5*$^{-/-}$ epidermis (n = 3) were γH2AX+ (p = 0.0001) (Figure 1P), suggesting impaired repair of DNA damage stemming from normal stem cell replication. Keratin 14 (Figures S3A–S3C) and loricrin (Figures S3D–S3F) stainings were normal.

Irx5 is expressed in proliferating HFSCs, and IRX5-binding motifs are enriched in HFSC super-enhancers

Noticing a roughened hair coat and mild hair loss in *Irx5*$^{-/-}$ mice at second telogen (Figure 2A), we next investigated the hair follicle role of IRX5. Distribution of auchene, awl, guard, and zigzag hair fibers was similar between *Irx5*$^{-/-}$ and *Irx5*$^{+/+}$ mice (Figures S3G–S3I), suggesting that *Irx5* plays a general role in hair follicle growth. The lower bulge of P20 *Irx5*$^{-/-}$ hair follicles had mild hypocellularity (Figure S3J), comparable to that of the IFE (Figure 1J).

RNA fluorescent *in situ* hybridization (RNA-FISH) showed that *Irx5* is expressed in the bulge region and adjacent secondary hair germ at P20 (early anagen) (Figures 2B and 2C). Expression increases at P28 and P32 (anagen) (Figure 2C), when *Irx5* RNA is abundant within the newly formed hair matrix (Figures 2B and S4A). This *in situ* localization of *Irx5* mRNA is consistent with publicly available transcriptomic databases, indicating high expression in HFSCs of P5 skin (Figure S4B) (Rezza et al., 2016) and outer bulge of telogen hair follicles (Figures S4C and S4D) (Joost et al., 2016, 2020). In these datasets, *Irx5* is expressed in the IFE, outer bulge, and fibroblast, but not in dermal papilla and dermal sheath. Together, these data indicate that *Irx5* expression is highest in proliferating epithelial cells of the hair follicle, both activated HFSCs and their progeny.

To test if IRX5 plays a direct role in HFSCs, we determined the IRX5 motif enrichment in previously published data on HFSC enhancers (Adam et al., 2015) and open chromatin (Adam et al., 2018). In HFSC super-enhancers, IRX5 motifs were enriched to a level comparable to the FOXP1, NFATc1, RFX2, and NFIB motifs, previously identified as top motifs for transcriptional regulators in HFSCs (Figure S5A) (Adam et al., 2018). IRX5 motifs were also enriched to levels higher than NFATC1, RFX2, and NFIB motifs in HFSC accessible chromatin regions (Figure S5B) (Adam et al., 2015). The prominence of predicted IRX5 motifs in active gene regulatory regions in HFSCs suggests that IRX5 directly regulates HFSC functions. As other IRX factors bind to similar motifs, other IRXs may also be involved in HFSC regulation.

Irx5 promotes hair follicle progenitor proliferation and hair growth initiation

Next, we studied if loss of IRX5 affects normal hair cycle timing. We shaved the backs of *Irx5*$^{-/-}$ (n = 4) and *Irx5*$^{+/+}$ (n = 5) littermates at P20 and monitored hair regrowth as an indicator of new growth phase (anagen) initiation (Figures 2D and 2E). *Irx5*$^{-/-}$ mice displayed significant delay (D = 0.392, p = 0.0017) in fur coverage compared with *Irx5*$^{+/+}$ littermates. *Irx5*$^{-/-}$ mice eventually entered anagen on P37, 7 days later than *Irx5*$^{+/+}$ littermates. Furthermore, hair growth in *Irx5*$^{-/-}$ mice initiated in an abnormal pattern, in a region in the center that propagated laterally. Typically, as in *Irx5*$^{+/+}$ littermate mice, first anagen starts in the shoulder region and propagates in a cranial-caudal direction (Wang et al., 2017).

Histology confirmed anagen initiation delay in *Irx5*$^{-/-}$ mice (Figure 2F). By P24, a clear delay in telogen-to-anagen transition was observed for *Irx5*$^{-/-}$ follicles. At P26 (mid-anagen for *Irx5*$^{+/+}$), *Irx5*$^{-/-}$ follicles displayed a thin epithelial column characteristic of anagen initiation. At P32, *Irx5*$^{+/+}$ follicles displayed regression of the epithelial column, indicative of catagen, whereas *Irx5*$^{-/-}$ skin displayed a dramatically thickened dermis containing the lower bulbs of hair follicles, suggesting the follicles had reached a delayed mid-anagen. At P46, *Irx5*$^{+/+}$ follicles exhibited telogen morphology, while *Irx5*$^{-/-}$ follicles were still in late anagen. At P56, *Irx5*$^{-/-}$ follicles eventually entered telogen.

To investigate the cause of the delayed anagen initiation in *Irx5*$^{-/-}$ hair follicles, we quantified proliferation by means of 2-h EdU pulses (Figures 2G–2J). At P20, proliferation was observed mainly in the secondary hair germs of hair follicles, with less proliferation in *Irx5*$^{-/-}$ hair germ (p = 0.0579) and bulge (p = 0.0418) compared with *Irx5*$^{+/+}$ (Figure 2H). At P28, *Irx5*$^{-/-}$ hair bulb cells continued to display significantly decreased proliferation ($p < 0.0001$), with 1% of *Irx5*$^{-/-}$ hair bulb cells staining EdU^{+} compared with 30% of *Irx5*$^{+/+}$ cells (Figure 2I). By mid-anagen (P32), proliferating cells were prominent in the matrix, with a higher proportion of EdU positivity in *Irx5*$^{-/-}$ compared with previous timepoints (Figure 2J). In sum, IRX5-deficient bulge cells are quiescent, and IRX5-deficient matrix cells display proliferative deficiency, indicating that IRX5 promotes proliferation of HFSCs and their progeny.

IRX5 promotes expression of cell cycle genes in HFSCs

We next investigated the mechanisms whereby IRX5 promotes HFSC activation. We isolated *Irx5*$^{-/-}$ and *Irx5*$^{+/+}$

(F) Representative H&E histology of *Irx5*$^{-/-}$ and *Irx5*$^{+/+}$ dorsal epidermis at the indicated time points.
(G) Representative EdU staining in P20, P28, and P32 hair follicles from *Irx5*$^{-/-}$ and *Irx5*$^{+/+}$ littermates. Scale bar represents 25 μm.
(H–J) Quantification of the percent of EdU^{+} cells between P20 *Irx5*$^{-/-}$ (n = 3) and *Irx5*$^{+/+}$ (n = 4) hair germ (p = 0.0579) and bulge (p = 0.0418) (H); P28 *Irx5*$^{-/-}$ (n = 2) and *Irx5*$^{+/+}$ (n = 2) hair bulb, $p < 0.0001$ (I); and P32 *Irx5*$^{-/-}$ (n = 2) and *Irx5*$^{+/+}$ (n = 2) hair bulb, p = 0.091 (J). Bu, bulge; HG, hair germ; DP, dermal papilla; Mx, matrix. p values obtained from two-sample t test at 95% CI.

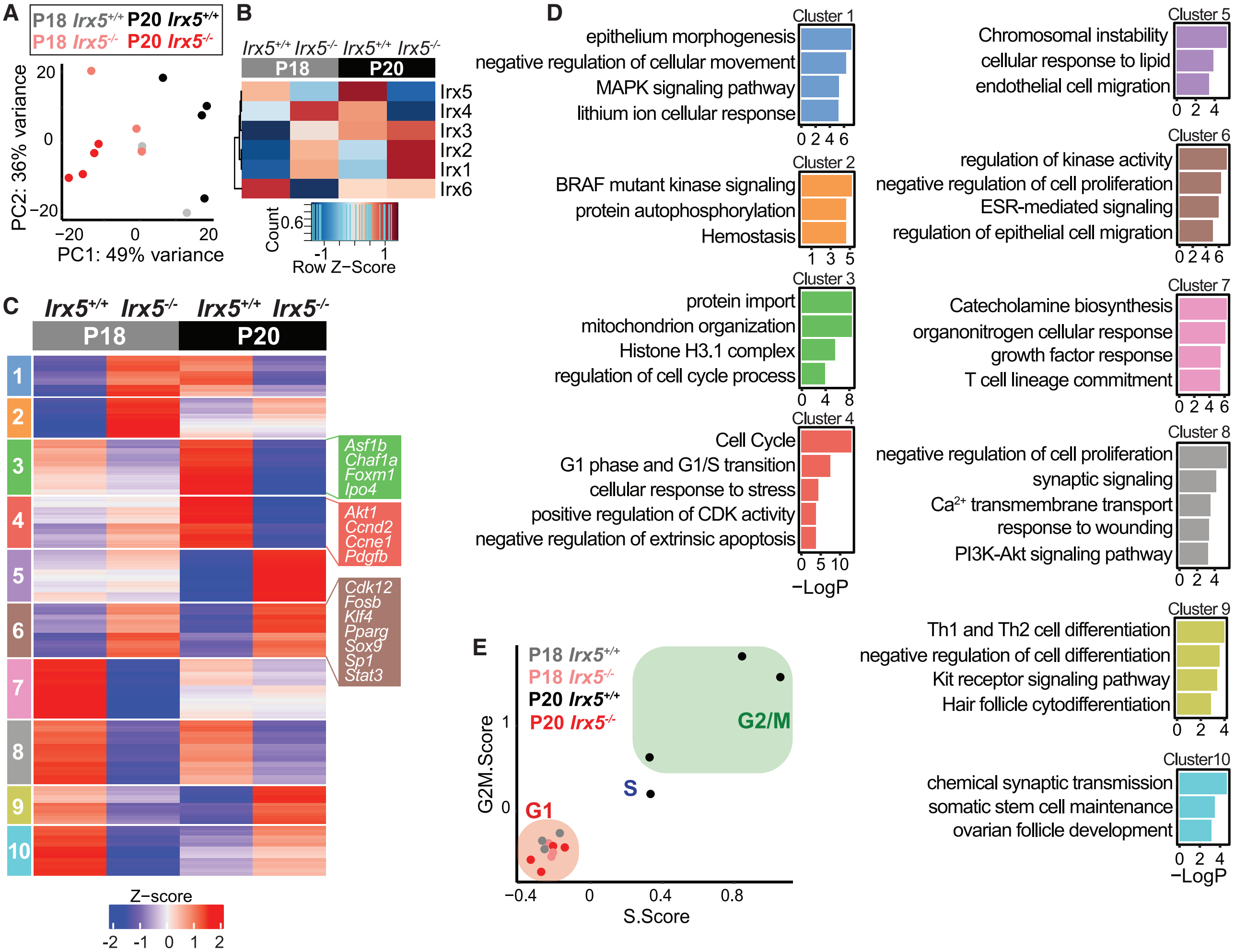

Figure 3. Altered cell cycle gene expression in *Irx5*$^{-/-}$ HFSCs

(A) PCA of HFSC gene expression in P18 *Irx5*$^{-/-}$ (n = 3) and *Irx5*$^{+/+}$ (n = 3) littermates along with P20 *Irx5*$^{-/-}$ (n = 4) and *Irx5*$^{+/+}$ (n = 4) littermates.

(B) Expression of *Irx1*, *Irx2*, *Irx3*, *Irx4*, *Irx5*, and *Irx6* in sorted P18 and P20 HFSCs.

(C) Hierarchical clustering of P18 and P20 HFSC gene expression data identified 10 distinct gene clusters.

(D) Top GO categories for each cluster in (C).

(E) Seurat cell cycle scoring was adapted to each replicate in bulk RNA-seq samples to identify the overall cell cycle stage of HFSCs isolated from each mouse.

HFSCs from two time points at the telogen/anagen transition, P18 (telogen) and P20 (early anagen in WT), and we subjected them to bulk RNA-seq analysis (Figure S6A). PCA segregated the samples by genotype across PC1, suggesting that gene expression differences between genotypes remain consistent across time points (Figure 3A).

In P18 HFSCs, *Irx5* and *Irx6* expression is high, whereas *Irx1, Irx2, Irx3, and Irx4* expression is low (Figure 3B). At P20, expression of *Irx1–5* increases, whereas *Irx6* expression decreases; *Irx5* is the highest expressing *Irx* at P20. Loss of *Irx5* results in upregulation of *Irx1–4* at P18 and *Irx1* and *Irx2* at P20 in HFSCs (Figure 3B), suggesting a negative-feedback regulatory loop among *Irx* genes. The increased expression of *Irx5* between P18 and P20 in HFSCs is consistent with the RNA-FISH data (Figure 2B) and its role in HFSC proliferation.

DEG analysis comparing P18 *Irx5*$^{-/-}$ (n = 3) and *Irx5*$^{+/+}$ (n = 3) HFSCs identified 2,259 downregulated and 630 upregulated genes in *Irx5*$^{-/-}$ HFSCs (Data S7). DEG analysis comparing P20 *Irx5*$^{-/-}$ (n = 4) and *Irx5*$^{+/+}$ (n = 4) HFSCs identified 1,668 downregulated and 1,464 upregulated genes in *Irx5*$^{-/-}$ HFSCs (Data S8). K-means clustering of these data revealed 10 distinct clusters (Figure 3C, Data S9), allowing us to define IRX5-affected genes at the

telogen-to-anagen transition. Genes in clusters 1 and 2 increase in expression from P18 to P20. Cluster 3 is of interest as its genes increase in expression from P18 to P20 and are downregulated in *Irx5*$^{-/-}$ at both P18 and P20 time points (Figure 3C). Cluster 3 is enriched in GO terms histone H3.1 complex organization and positive regulation of cell cycle (Figure 3D). Histone H3.1 complex organization genes include *Chaf1a*, *Asf1b,* and *Ipo4*. CHAF1A is a critical subunit of histone H3.1, a transient histone that is required for DNA replication and DNA repair (Tagami et al., 2004). Downregulation of these critical cell cycle progression histone subunits in *Irx5*$^{-/-}$ HFSCs occurs both at P18 and P20, suggesting that IRX5 may be a constitutive regulator of histone subunits.

Cluster 4, which is enriched in cell cycle progression genes, also shows upregulation between P18 and P20; these gene are downregulated in *Irx5*$^{-/-}$ HFSCs at P20 (Figures 3C and 3D). Cluster 5, which is downregulated between P18 and P20, contains genes that are upregulated in P18 and P20 *Irx5*$^{-/-}$ compared with *Irx5*$^{+/+}$ HFSCs. This cluster contains genes involved in chromosomal and microsatellite instability. Cluster 6 gene expression remained static between P18 and P20 *Irx5*$^{+/+}$ HFSCs, suggesting that gene expression in this cluster does not change in early anagen. At both P18 and P20, cluster 6 genes are upregulated in *Irx5*$^{-/-}$ HFSCs, suggesting that IRX5 represses these genes regardless of hair cycle stage (Figure 3D). Cluster 6 is enriched in genes involved in negative regulation of cell proliferation including *Klf4*, a growth arrest factor that induces the expression of CDKN1A (Chen et al., 2003). Cluster 7 contains genes upregulated at P18 compared with P20; these genes are downregulated in P18 *Irx5*$^{-/-}$ HFSCs. This cluster contains growth factor response genes including FGF6. Cluster 8, which contains genes involved in PI3K-Akt signaling, remains highly expressed across P18 and P20 but is downregulated in *Irx5*$^{-/-}$ HFSCs. Cluster 9 and 10 genes, which are enriched in factors related to differentiation, are downregulated between P18 and P20, and they are differentially expressed in P18 and P20 *Irx5*$^{-/-}$ HFSCs.

As *Irx5*$^{+/+}$ mice have progressed to early anagen at P20 while *Irx5*$^{-/-}$ mice remain at telogen, gene expression differences between the genotypes at P20 may be indirect. But several gene expression clusters show a consistent change between genotypes at P18 and P20, suggesting that those genes represent IRX5-regulated genes. These include clusters 3, 6, and 8, which identify perturbed epithelial proliferation, tyrosine kinase receptor signaling, and apoptosis (Figure S6B). Transcriptional regulatory relationship analysis of these genes found transcription factors related to proliferation like MYC, NFKB, E2F, and SP1 (Figure S6C). Gene-disease association analysis of the combined clusters identified metastatic cancers such as epithelioma, ovarian carcinoma, as well as xeroderma pigmentosum, a nucleotide excision repair disease in skin (Figure S6D).

Seurat's (Stuart et al., 2019) cell cycle scoring function predicted that P18 and P20 *Irx5*$^{+/+}$ HFSCs are quiescent and proliferating, respectively (Figure 3E). By contrast, P20 *Irx5*$^{-/-}$ HFSCs are predicted to be quiescent (Figure 3E), consistent with the EdU data on P20 *Irx5*$^{-/-}$ hair follicles (Figure 2G). Collectively, these findings indicate that IRX5 promotes proliferation of HFSCs, most likely by activating and repressing, respectively, positive and negative regulators of cell proliferation and by upregulating expression of histone subunits necessary for cell cycle progression.

IRX5 promotes expression of DNA damage repair factors and repression of *Fgf18*

DEG analysis of P20 *Irx5*$^{-/-}$ and *Irx5*$^{+/+}$ HFSCs (Data S8) identified *Brca1* and *Fgf18*, respectively, as top downregulated and upregulated genes in *Irx5*$^{-/-}$ HFSCs (Figure 4A). Downregulated genes are significantly enriched in cell cycle checkpoint and DNA repair GO categories (Figure 4B), whereas upregulated genes are enriched in positive regulation of cell death GO categories (Figure 4C). The DNA damage repair genes that are consistently downregulated in *Irx5*$^{-/-}$ HFSCs include *Brca1*, *Bard1*, *Mlh1*, *Fancd2*, *Rad51,* and *Exo1*—genes that are involved in mismatch repair and homologous recombination (Figure 4D). These DNA damage repair genes are normally upregulated from P18 to P20, suggesting that they are necessary as HFSCs start proliferating. These genes are downregulated in *Irx5*$^{-/-}$ HFSCs at P18 and P20, suggesting that IRX5 may be a direct regulator of these critical DNA damage repair genes. *Fgfs* are downregulated in *Irx5*$^{-/-}$ HFSCs at P18 and P20 with the exception of *Fgf18* and *Fgf21* (Figure 4E), which are upregulated at P20.

Brca1, *Bard1*, and *Rad51*, all of which are downregulated in *Irx5*$^{-/-}$ HFSCs at P18 and P20, are indispensable for homologous recombination (Zhao et al., 2017). *Brca1*-deficient mice have less hair growth, with existing *Brca1*$^{-/-}$ hair follicles displaying increased DNA damage and p53-dependent apoptosis in HFSCs (Sotiropoulou et al., 2013). Consistently, Ingenuity Pathway Analysis predicted an increase in DNA damage, activated ATM/MAPK pathway, stabilized TP51, activated CDKN1A, and inhibited cell proliferation in *Irx5*$^{-/-}$ HFSCs (Figure 4F).

IRX5 modifies open chromatin regions in HFSCs

In our P18 and P20 RNA-seq datasets, we identified altered histone regulation in *Irx5*$^{-/-}$ HFSCs (Figure 3D), suggesting altered epigenetic landscape in *Irx5*$^{-/-}$ HFSCs. We, therefore, conducted assay for transposase-accessible chromatin (ATAC) sequencing on fluorescence-activated cell sorting-isolated HFSCs from the back skins of *Irx5*$^{-/-}$ (n = 2) and

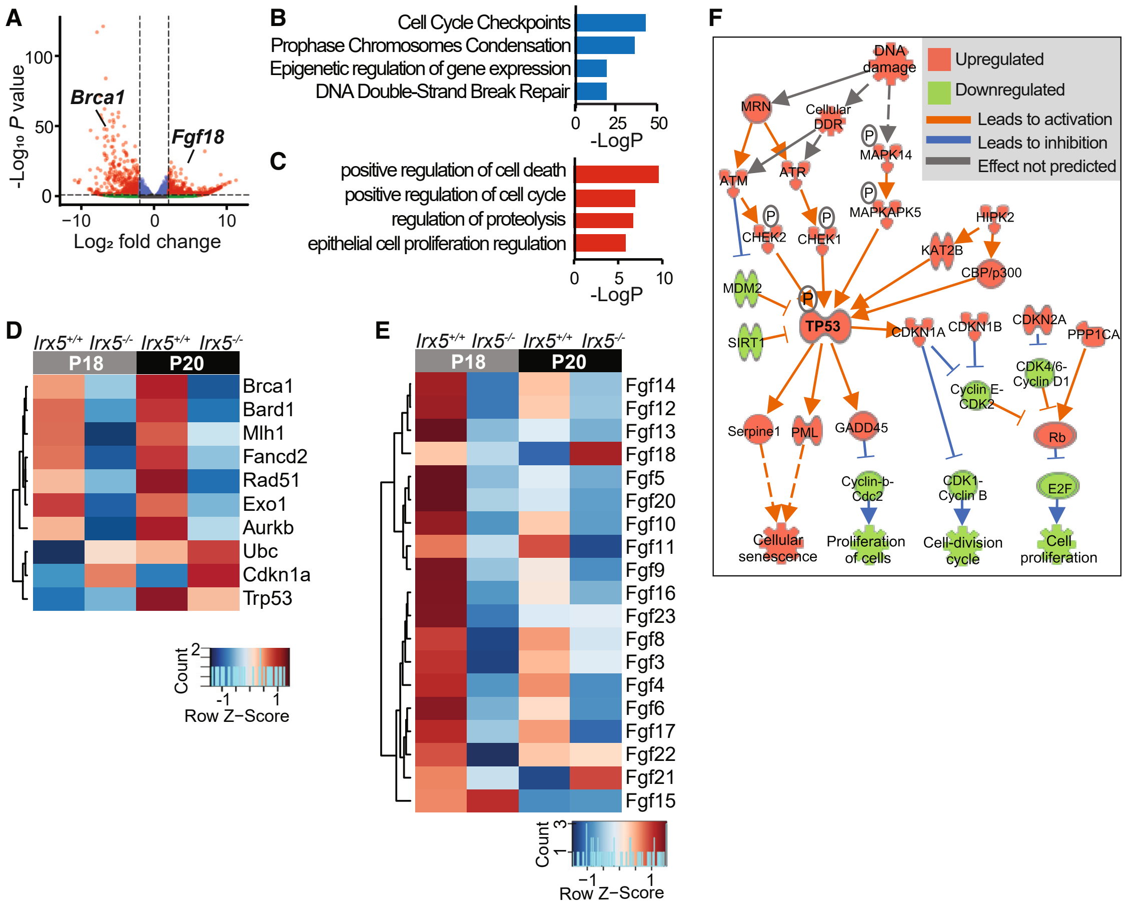

Figure 4. Defective DNA repair gene expression in P20 *Irx5*$^{-/-}$ HFSCs

(A) Volcano plot of DEGs between P20 *Irx5*$^{-/-}$ and *Irx5*$^{+/+}$ HFSCs. *Brca1* is downregulated and *Fgf18* is upregulated in *Irx5*$^{-/-}$ HFSCs.
(B) GO enrichment of downregulated genes in (A).
(C) GO enrichment of upregulated genes in (A).
(D) Heatmap of averaged expression of genes involved in DNA damage repair in HFSCs under the indicated conditions.
(E) Heatmap of averaged expression of *Fgf* in HFSCs under the indicated conditions.
(F) Ingenuity Pathway Analysis of the gene expression data in (A) predicts perturbed DNA damage in P20 *Irx5*$^{-/-}$ HFSCs.

Irx5$^{+/+}$ (n = 2) mice at P20. We identified on average 20,970 peaks in *Irx5*$^{+/+}$ and 32,692 peaks in *Irx5*$^{-/-}$ HFSCs that were proportionally similar in their locations in introns, exons, and promoters (Figure S7A). The majority of differentially open chromatin regions were unique to *Irx5*$^{-/-}$ HFSCs (Figure 5A); there were 4,277 unique peaks in the *Irx5*$^{-/-}$ and five unique peaks in *Irx5*$^{+/+}$ HFSCs. Motif analysis of *Irx5*$^{+/+}$ HFSC open chromatin revealed significant enrichment for IRX5 as well as for known HFSC transcription factors NFATc1, LHX2, and JUND (Figure S7B), consistent with public data on open chromatin in HFSCs (Yang et al., 2017) (Figures S5A and S5B).

The Genomic Regions Enrichment of Annotations Tool found that *Irx5*$^{+/+}$ HFSC open chromatin regions are enriched in mouse phenotypes related to epidermal function (Figure 5B). By contrast, *Irx5*$^{-/-}$ differential open chromatin contains mouse phenotype ontology related to abnormal DNA repair and early cellular replicative senescence, demonstrating that the majority of the aberrant unique open chromatin in *Irx5*$^{-/-}$ HFSCs regulate genes related to DNA repair (Figure 5C).

We then overlapped *Irx5*$^{+/+}$ HFSC open chromatin regions with consistent DEGs from P18 and P20 *Irx5*$^{-/-}$ HFSCs, using Binding and Expression Target Analysis (BETA) (Figures 5D–5J). BETA scored the association of each perturbed gene in relation to its genomic distance from the *Irx5*$^{+/+}$ HFSC open chromatin and ranked each DEG in terms of its likeliness to be involved in normal HFSC biology. BETA found that the *Irx5*$^{-/-}$ upregulated genes are involved in cell adhesion, apoptosis, histone H3 methylation, and DNA damage response (Figure 5E), while the majority of downregulated genes are involved in

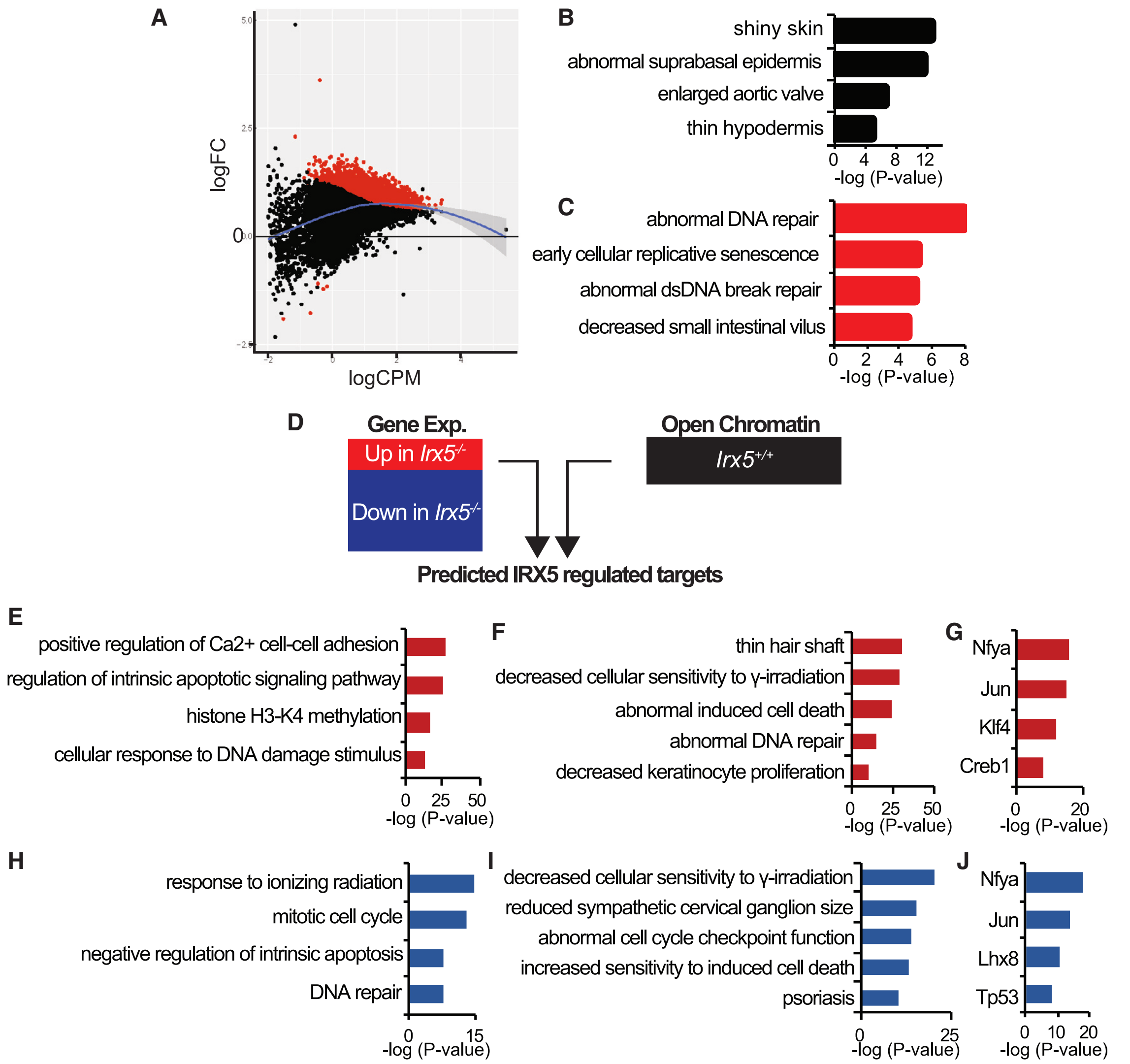

Figure 5. IRX5 maintains closed chromatin at cell cycle and DNA repair genes in HFSCs

(A) ATAC-seq on *Irx5*$^{+/+}$ (n = 2) and *Irx5*$^{-/-}$ (n = 2) P20 HFSCs identified differential open chromatin regions. The majority of differential chromatin regions represent accessible chromatin in *Irx5*$^{-/-}$ HFSCs.
(B) Top mouse phenotype ontology categories associated with *Irx5*$^{+/+}$ HFSC open chromatin regions.
(C) Top mouse phenotype ontology categories associated with *Irx5*$^{-/-}$ HFSC open chromatin regions.
(D) Schematic of Binding and Expression Target Analysis with differential gene expression and open chromatin data: DEGs identified from P18 P20 cluster analysis are combined with normal HFSC open chromatin to predict IRX5-regulated targets.
(E) GO of differential open chromatin regions associated with upregulated genes in P20 *Irx5*$^{-/-}$ HFSCs.
(F) Mouse phenotype ontology of differential open chromatin regions associated with upregulated genes in P20 *Irx5*$^{-/-}$ HFSCs.
(G) Enriched transcription factor binding motifs in differential open chromatin regions associated with upregulated genes in P20 *Irx5*$^{-/-}$ HFSCs.
(H) GO of differential open chromatin associated with downregulated genes in P20 *Irx5*$^{-/-}$ HFSCs.
(I) Mouse phenotype ontology of differential open chromatin associated with downregulated genes in P20 *Irx5*$^{-/-}$ HFSCs.
(J) Enriched motif of differential open chromatin associated with downregulated genes in P20 *Irx5*$^{-/-}$ HFSCs.

mitotic cell cycle processes (Figure 5H). Furthermore, mouse phenotype ontology of these genomic regions of *Irx5*$^{-/-}$ upregulated genes display thin hair shaft, abnormal cell death, and abnormal DNA repair (Figure 5F), while *Irx5*$^{-/-}$ downregulated genes display abnormal cell cycle checkpoint function (Figure 5I).

The open chromatin regions near the upregulated (Figure 5G) and downregulated (Figure 5J) DEGs are both

enriched in NFYA motifs. Furthermore, *Irx5*$^{-/-}$ unique open chromatin regions (Figure 5I) are enriched in NFYA motifs (Figure S7C). NFYA represses cell cycle progression genes (Yun et al., 2003), suggesting that IRX5 prevents the epigenetic repression of these cell cycle progression genes.

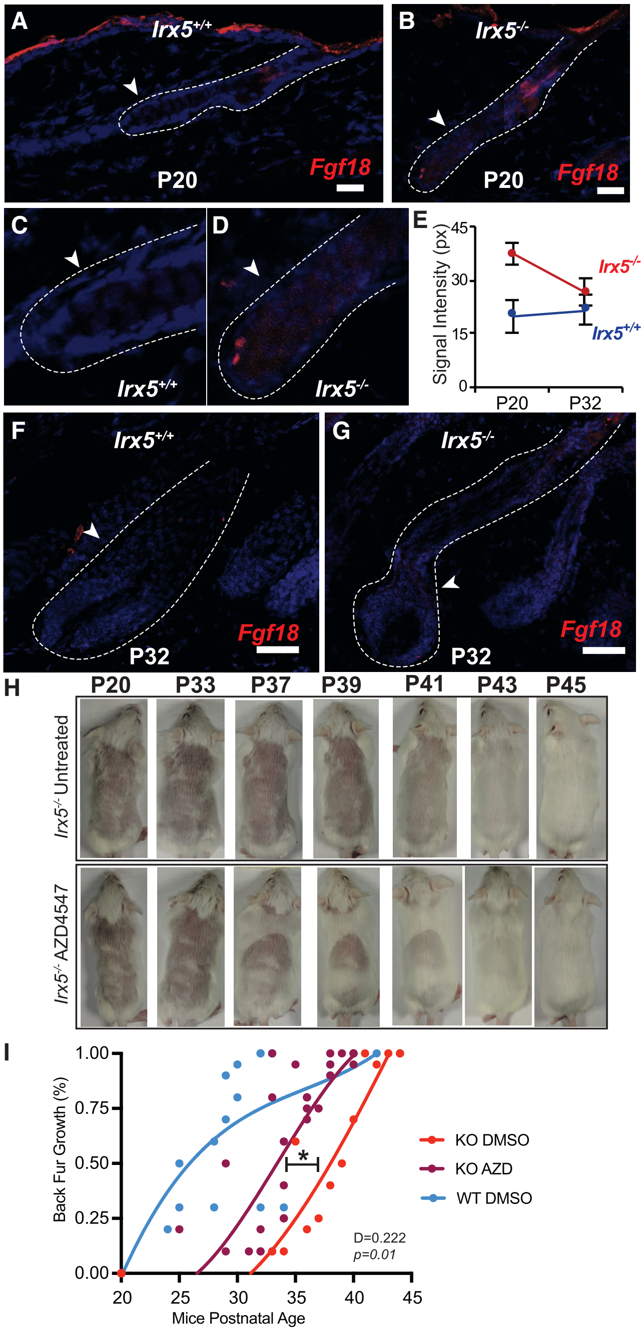

Figure 6. IRX5 suppresses the expression of *Fgf18* in the hair follicle bulge during early anagen

(A and B) RNA-FISH of *Fgf18* in P20 hair follicles; arrows point to lower bulge. Scale bar represents 10 μm.

(C and D) Higher magnification images of the lower bulge.

(E) Quantification of pixel intensity of lower bulge *Fgf18* signal in hair follicles from p20 *Irx5*$^{+/+}$ ($n = 3$) and *Irx5*$^{-/-}$ ($n = 4$) ($p < 0.0001$); and p32 *Irx5*$^{+/+}$ ($n = 3$), *Irx5*$^{-/-}$ ($n = 3$) mice.

(F and G) RNA-FISH of *Fgf18* in P32 hair follicles; arrows point to lower bulge. Scale bar represents 10 μm.

(H) Representative hair growth after shaving at P20 in *Irx5*$^{-/-}$ mice treated with DMSO or AZD4547.

(I) Quantification of hair growth through first telogen and anagen in DMSO-treated *Irx5*$^{+/+}$ ($n = 7$), DMSO-treated *Irx5*$^{-/-}$ ($n = 9$), and AZD4547-treated *Irx5*$^{-/-}$ mice ($n = 11$). Partial rescue of hair phenotype was observed in AZD4547 treated *Irx5*$^{-/-}$ mice ($D = 0.222$, $p = 0.0106$). Statistical analysis conducted with two-sided Kolmogorov-Smirnov test.

IRX5 represses FGF-induced HFSC quiescence

Fgf18 is highly upregulated in *Irx5*$^{-/-}$ HFSCs at P20 (Figure 4A), and although it is not differentially regulated at P18, *Fgf18* levels specifically rise from P18 to P20 in *Irx5*$^{-/-}$ HFSCs (Figure 4E). *Fgf18* is a critical downstream target of *Foxp1* that maintains HFSC quiescence and inhibits anagen initiation (Kimura-Ueki et al., 2012; Leishman et al., 2013). To validate the RNA-seq data and to locate the expression of *Fgf18*, we conducted RNA-FISH on hair follicles at various stages of early (Figures 6A–6D) and late (Figures 6F and 6G) anagen. At P20 and P32, *Fgf18* was not visible in *Irx5*$^{+/+}$ hair follicles (Figures 6A, 6C, and 6F), consistent with previous findings (Kimura-Ueki et al., 2012). By contrast, *Irx5*$^{-/-}$ hair follicles express detectable *Fgf18* levels at P20 ($p < 0.0001$) (Figures 6B, 6D, and 6E).

To determine whether upregulation of FGF could mediate the delayed anagen initiation in *Irx5*$^{-/-}$ mice, we inhibited the downstream signaling of FGF with the pan-FGFR inhibitor AZD4547 (Kawano et al., 2016). *Irx5*$^{-/-}$ mice were treated with 10 μM/g AZD4547 ($n = 11$) or 1% DMSO saline ($n = 9$) every two days from P20 to P46. *Irx5*$^{+/+}$ littermates were treated with 1% DMSO saline ($n = 7$) as additional controls. AZD4547 partially rescued hair growth with a 4-day acceleration of back fur recovery in the AZD4547-treated *Irx5*$^{-/-}$ group compared with the DMSO-treated *Irx5*$^{-/-}$ group ($D = 0.222$, $p = 0.0106$) (Figures 6H and 6I). AZD4547 treatment, however, did not fully rescue hair growth ($D = 0.195$, $p = 0.06$) (Figure 6I). These results suggest that upregulation of FGF mediates part of the anagen delay due to DNA damage in *Irx5*$^{-/-}$ mice.

IRX5 activates *Brca1* expression in HFSCs

The gene expression and epigenetic experiments described above predict that IRX5 controls the cell cycle and DNA damage repair in HFSCs. To validate that *Brca1* expression

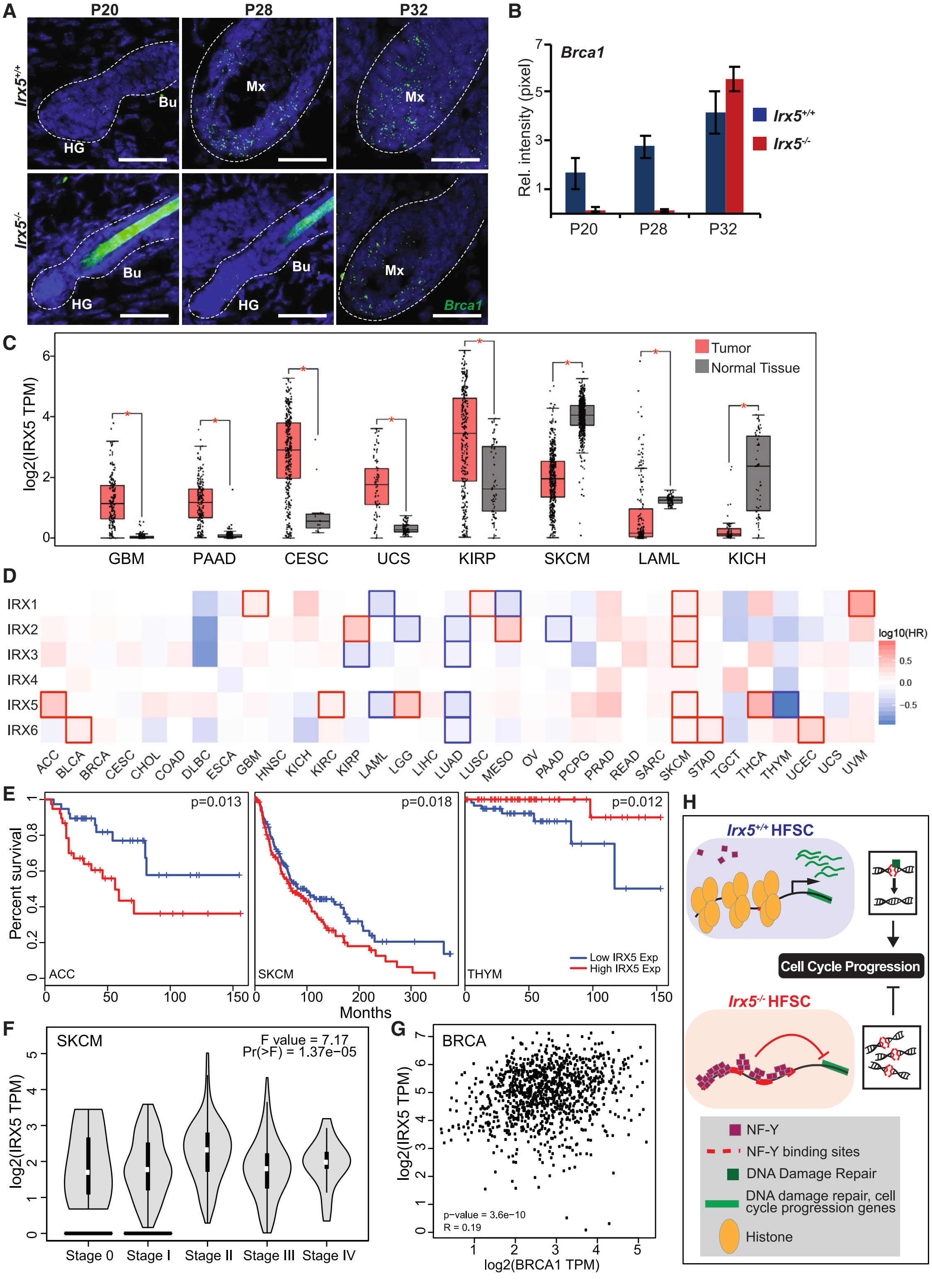

(legend on next page)

is downregulated in *Irx5*$^{-/-}$ hair follicles, we used RNA-FISH to analyze its expression at P20, P28, and P32 (Figure 7A). *Brca1* is expressed in the P20 hair germs of *Irx5*$^{+/+}$ hair follicles. By P28 and P32, *Brca1* expression markedly increases in the *Irx5*$^{+/+}$ matrix, primarily in the lower bulge. By comparison, *Irx5*$^{-/-}$ hair follicles expressed *Brca1* at low levels at P20 ($p < 0.001$) and P28 ($p < 0.001$) (Figure 7B). By P32, *Brca1* is expressed in the lower bulge of *Irx5*$^{-/-}$ hair follicles (Figure 7B). Analysis of comparable follicles at the telogen-to-anagen transition, P28 *Irx5*$^{-/-}$ and P20 *Irx5*$^{+/+}$, found higher *Brca1* expression in *Irx5*$^{+/+}$ than *Irx5*$^{-/-}$ hair germs ($p < 0.0001$) (Figure 7B). Together with the transcriptomics data showing decreased *Brca1* expression in HFSCs at both P18 and P20 (Figure 4D), these experiments indicate that *Irx5*$^{-/-}$ HFSCs have decreased BRCA1 expression.

Role of IRX in carcinogenesis is tumor dependent

As our data indicate that *Brca1* expression is Irx5 dependent, we characterized the expression of *IRX5* and the relationship between *IRX5* and *BRCA1* in tumorigenesis, using publicly available TCGA (The Cancer Genome Atlas) data. *IRX5* is overexpressed in the majority of tumor types, although some tumor types have decreased *IRX5* expression (Figure 7C) (Tang et al., 2017). Survival analysis of *IRX1–IRX6* expression across TCGA cancer types suggests a mostly unfavorable prognosis with higher *IRX* expression, although in some tumors high *IRX* expression confers better prognosis (Figures 7D and 7E). Higher expression of *IRX1*, *IRX2*, *IRX3*, *IRX5*, and *IRX6* all display statistically significant unfavorable prognosis for skin cutaneous melanoma (Figure 7D). Analysis of *IRX5* expression across the clinical stages of skin cutaneous melanoma indicates a statistically significant correlation between higher *IRX5* expression and advanced pathological tumor stage (Figure 7F). Consistent with *BRCA1* being a downstream target of IRX5, there was significant correlation between *BRCA1* and *IRX5* gene expression in breast-invasive carcinoma (Figure 7G), suggesting that the regulatory relationship between IRX5 and BRCA1 extends beyond epidermal keratinocytes.

In sum, we demonstrate that IRX5 is required for DNA damage repair and cell cycle progression in HFSCs at the telogen-to-anagen transition and in epithelial progeny of HFSCs in early anagen hair follicles. IRX5 operates through multiple mechanisms. First, IRX5, directly or indirectly, upregulates BRCA1 and other DNA repair factors. Second, IRX5 regulates histone expression and suppresses open chromatin regions that negatively regulate transcription of DNA damage repair and cell cycle progression genes. Third, IRX5, through its promotion of DNA repair, contributes to the downregulation of quiescence-maintaining factors, reducing inhibitory signals for the telogen-to-anagen transition (Figure 7H).

Figure 7. IRX5 has a tissue-dependent role in carcinogenesis

(A) RNA-FISH staining of *Brca1* in p20, p28, and p32 follicles from *Irx5*$^{-/-}$ and *Irx5*$^{+/+}$ mice. Scale bar represents 25 μm.

(B) Quantification of *Brca1* signal intensity in p20 *Irx5*$^{-/-}$ (n = 2), p20 *Irx5*$^{+/+}$ (n = 2) $p < 0.001$; p28 *Irx5*$^{-/-}$ (n = 2), p28 *Irx5*$^{+/+}$ (n = 2) $p < 0.001$; p32 *Irx5*$^{-/-}$ (n = 2), p32 *Irx5*$^{+/+}$ (n = 2) hair follicles. Bu, bulge; HG, hair germ; DP; dermal papilla; Mx, matrix. p values obtained from two-sample t test at 95% CI.

(C–G) TCGA data were analyzed using GEPIA2. Statistical analysis conducted with one way ANOVA.

(C) *IRX5* expression in tumors compared with its normal tissue.

(D) Survival heatmap displaying prognostic impacts of *IRX1–IRX6* expression among all TCGA cancer types. Red and blue blocks denote higher and lower risks, respectively. Framed rectangle identifies statistically significant survivability: higher expression of *IRX* is unfavorable in 17 instances while favorable in 11 instances.

(E) Representative statistically significant survival analysis of IRX5 expression.

(F) *IRX5* expression across the clinical stages of skin cutaneous melanoma.

(G) Correlation analysis of *IRX5* and *BRCA1* expression among breast-invasive carcinoma.

(H) Repressive NF-Y binding sites lie near genes that promote cell proliferation and DNA damage repair. In *Irx5*$^{+/+}$ HFSCs, repressive NF-Y binding sites are closed, which allows for the transcription of genes involved in DNA damage repair and cell cycle progression. If DNA damage occurs prior to replication, it is repaired. In contrast, the NF-Y binding sites in *Irx5*$^{-/-}$ HFSCs are open, thus repressing the expression of DNA damage repair and cell cycle progression genes. Consequently, accumulation of DNA damage occurs, which triggers cellular senescence. Abbreviations are as follows: ACC, adrenocortical carcinoma; BLCA, bladder urothelial carcinoma; BRCA, breast-invasive carcinoma; CESC, cervical squamous cell carcinoma and endocervical adenocarcinoma; CHOL, cholangio carcinoma; COAD, colon adenocarcinoma; DLBC, lymphoid neoplasm diffuse large B cell lymphoma; ESCA, esophageal carcinoma; GBM, glioblastoma multiforme; HNSC, head and neck squamous cell carcinoma; KICH, kidney chromophobe; KIRC, kidney renal clear cell carcinoma; KIRP, kidney renal papillary cell carcinoma; LAML, acute myeloid leukemia; LGG, brain lower grade glioma; LIHC, liver hepatocellular carcinoma; LUAD, lung adenocarcinoma; LUSC, lung squamous cell carcinoma; OV, ovarian serous cystadenocarcinoma; PAAD, pancreatic adenocarcinoma; PCPG, pheochromocytoma and paraganglioma; PRAD, prostate adenocarcinoma; READ, rectum adenocarcinoma; SARC, sarcoma; SKCM, skin cutaneous melanoma; STAD, stomach adenocarcinoma; TGCT, testicular germ cell tumors; THCA, thyroid carcinoma; THYM, thymoma; UCEC, uterine corpus endometrial carcinoma; UCS, uterine carcinosarcoma.

DISCUSSION

Whereas previous studies on IRX5 have focused on its role as a developmental gene regulator (Cheng et al., 2005; Gaborit et al., 2012), our findings point to a role in stem cell proliferation. We link cell cycle functions with the DNA damage response and suggest that the role of IRX5 in DNA damage repair could be an overlooked mechanism in the emerging link between IRX5 and carcinogenesis (Huang et al., 2018; Zhu et al., 2020).

IRX5 promotes cell cycle progression and DNA damage repair in HFSCs and their progeny

Although previous studies, primarily in cancer cells, have characterized the proliferative role of IRX5 (Liu et al., 2016; Son et al., 2021; Sun et al., 2020; Zhu et al., 2020), little is known about its role in DNA damage. Accumulated DNA damage in long-lived stem cells causes cancer or aging-related degeneration. Adult stem cells address DNA damage with diverse methods. HFSCs are resistant to DNA damage-induced apoptosis in part due to elevated expression of anti-apoptosis factor BCL-2 and enhanced non-homologous end joining (NHEJ) DNA repair (Sotiropoulou et al., 2010). *Irx5*$^{-/-}$ HFSCs displayed diminished expression of NHEJ mediators H2B histones and TDP1, a phosphodiesterase necessary for efficient NHEJ (Li et al., 2017). We also identified other DNA damage repair factors perturbed in *Irx5*$^{-/-}$ HFSCs, MLH1, EXO1, and RAD51, which are involved in nucleotide excision repair (NER) and homologous recombination (HR). While there are no accounts of these DNA damage repair mechanisms in HFSCs, NER and HR are the preferential DNA damage repair pathways of proliferating adult stem cells similar to activated HFSCs (Hua et al., 2012).

These DNA damage repair factors are downstream of key regulators FOXM1 and BRCA1, which are both downregulated in *Irx5*$^{-/-}$ HFSCs (Figures 3C, 4D and 7A). FOXM1 is primarily a direct regulator of factors involved in single-stranded DNA break repair such as NER (Khongkow et al., 2014). BRCA1 promotes HR over NHEJ (Wu et al., 2010). Conditional knockout of *Brca1* in the epidermis leads to hair loss with rapidly degenerating hair follicles due to increased apoptosis (Sotiropoulou et al., 2013). While not as severe, *Irx5*$^{-/-}$ mice display similar hair findings as *Brca1*$^{-/-}$ mice with downregulated DNA damage repair genes (Figure 4D) in HFSCs, as well as delayed hair growth.

IRX5 controls the chromatin landscape in HFSCs

In *Irx5*$^{-/-}$ HFSCs, we identified multiple open chromatin regions near DNA damage repair genes (Figure 5C). These new open chromatin regions are enriched for motifs for NFYA (Figure S7C), a transcription factor that acts as both transcriptional activator and repressor. In response to DNA damage, NF-Y mediates transcriptional inhibition of cyclin expression with cell cycle arrest through P53-dependent HDAC4 histone deacetylation (Basile et al., 2006). Our data suggest that loss of IRX5 leads to increased chromatin access for NF-Y with inhibition of cyclin expression.

Previous studies have suggested that IRX5 is a direct epigenetic regulator with both transcriptional activating and repressing functions (Bonnard et al., 2012; Costantini et al., 2005) depending on signaling or cellular context. The IRX5 IRO box is known to associate with m-BOP and to recruit HDAC, thus promoting chromatin condensation and silencing of nearby genes (Costantini et al., 2005). In *Irx5*$^{-/-}$ HFSCs, we identified downregulation of histone H3 subunits (Figure 3D), which are required for DNA replication and repair (Tagami et al., 2004). FOXM1, downregulated in all NHEK *IRX* knockdown (Figure 1C) and in *Irx5*$^{-/-}$ HFSCs (Figure 3C), is also a critical cell cycle regulator that co-localizes near NF-Y binding sites through protein-protein interaction with the MMB complex to bypass senescence (Chen et al., 2013).

IRX5 promotes proliferation and DNA repair in the interfollicular epidermis

The role of IRX5 in promoting DNA repair is not limited to HFSCs as we observed increased DNA damage (Figure 1P) and decreased proliferation (Figure 1M) in *Irx5*$^{-/-}$ EpiSCs. We also observed cell cycle progression defects with IRX5 loss of function in cultured NHEKs (Figures 1D and S1C), suggesting that the epidermal cell proliferation role of IRX5 is conserved from mice to humans.

In contrast to the marked hair follicle defect, we did not observe defects in the function of the interfollicular epidermis (IFE) of *Irx5*$^{-/-}$ mice; there is only a subtle hypocellularity of the basal layer (Figure 1J). The reason for this dichotomy, which was also observed in studies on *Brca1* null mice (Sotiropoulou et al., 2013) and an ectopic Bcl-2 mouse model (Geueke et al., 2021), is unknown. Studies on chronic low UV irradiation of mouse epidermis have found that basal IFE keratinocytes capable of NER accumulate DNA damage and remain viable (Nijhof et al., 2007), suggesting that basal keratinocytes continue to maintain tissue homeostasis despite unrepaired DNA damage. Furthermore, the IFE is maintained as a continuous sheet by competing epidermal clones whereby DNA damaged epidermal clones are likely outcompeted by neighboring genetically intact clones. Hair follicles on the other hand rely on a limited pool of synchronous stem cells, requiring immediate repair upon DNA damage. In addition, the very high proliferation rate of hair follicle progenitor cells in early anagen may make the hair follicle more sensitive to DNA damage than the IFE.

IRX5 promotes anagen initiation through repression of *Fgf18*

Several mechanisms have been found to regulate HFSC quiescence and anagen initiation (Geyfman et al., 2015), including the secreted factor FGF18. FGF18 is an autocrine factor expressed in bulge and bulge-derived cells with paracrine activity on nearby HFSCs (Hsu et al., 2011). In addition to its physiologic role, FGF18 has been shown to be upregulated upon radiation-induced hair follicle damage, where it is presumed to induce cell cycle arrest to allow for DNA repair in HFSCs (Kawano et al., 2016). Consistent with these ideas, we observed increased *Fgf18* expression in $Irx5^{-/-}$ HFSCs (Figures 4A and 6E). Pan-FGF receptor kinase inhibition partially rescued the anagen delay in $Irx5^{-/-}$ mice (Figures 6H and 6I). While the FGFR inhibitor could also perturb other FGF signaling and while P20 $Irx5^{-/-}$ HFSCs differentially express other FGFs (Figure 4E), we believe that FGF18 is one of the central contributors to $Irx5^{-/-}$ anagen delay as it has been previously identified as a key factor that prolongs telogen (Kimura-Ueki et al., 2012).

IRX5 in human disease

Data in the Genome Aggregation Database (gnomAD) indicate that *IRX5* is intolerant to loss of function variants in humans. On the other hand, missense mutations in the highly conserved DNA-binding domain of IRX5 are responsible for the rare autosomal recessive Hamamy syndrome (Mégarbané et al., 2021). This syndrome, which is characterized by cranio-facial abnormalities, osteopenia, sensory hearing loss, and mental retardation, has been linked to decreased stromal cell-derived factor 1 expression and abnormal migration of neural crest cells (Bonnard et al., 2012). Recently, sparse hair was noted in patients affected by Hamamy syndrome (Mégarbané et al., 2021), a phenotype that could link to the hair follicle findings described in our study. Based on our findings, a role for IRX5 in cell cycle and DNA damage responses in epithelia should be considered as a disease mechanism in some of the Hamamy syndrome manifestations.

Although *IRX5* may suppress tumorigenesis, multiple studies have found *IRX5* to be upregulated in cancers (Huang et al., 2018; Sun et al., 2020), suggesting a pro-oncogenic role. Our results here suggest that promotion of DNA repair and suppression of apoptosis should be explored as mechanisms linking IRX5 to tumor progression.

EXPERIMENTAL PROCEDURES

Resource availability

Corresponding author

Requests should be directed to corresponding author Bogi Andersen (bogi@uci.edu).

Materials availability

No new materials were generated in this study.

Data and code availability

Sequencing data are available under GEO Super-Series GSE202076.

Mice

$Irx5^{-/-}$ mice and their genotyping were described previously. All mouse experiments were conducted on sex-matched littermates in accordance with University of California, Irvine Institutional Animal Care and Use Committee (Protocol No. AUP-19-012).

Tissue and cell isolation

P18 and P20 back skin was dissected and the epidermis isolated. Ly-6A/E^{-} CD34^{+} CD49f^{+} HFSCs were isolated for bulk RNA-seq and ATAC-seq.

Cell culture

NHEKs were grown in keratinocyte serum free media supplemented with epidermal growth factor and bovine pituitary extract. 30 nM pooled siRNAs was transfected into semi-confluent monolayers. 12 hours after transfection, 1.8 mM Ca^{2+} was added to the medium to induce differentiation. RNA lysate was collected 72 h after transfection.

Bulk RNA sequencing

Library preparation was done with Illumina TrueSeq library preparation kit, and single-end Illumina HiSeq 2500 sequencing was performed.

ATAC-seq

Nuclei were incubated in 50 uL of Tn5 transposition buffer for 30 min at 37°C. Library preparation with Illumina TrueSeq library preparation kit and paired-end Illumina HiSeq 2500 sequencing (80 million reads per sample) was performed at the University of California, Irvine High Throughput Genomics Facility. Motif analysis was conducted with MEME Suit 5.5.0 using predicted IRX5 motif (JASPAR, 2020 #PH0086.1).

RNA and protein detection

For immunofluorescence localization of protein, fresh tissue samples were harvested and embedded in optimal cutting temparture (OCT) compound. For RNA-FISH, fresh frozen 10-μM-thick OCT sections were processed and stained using RNAscope Multiplex Fluorescent Detection Kit v1.

Details on mouse work, cell culture, RNA and protein detection in tissues, RNA-seq, and ATAC-seq are provided in the supplemental experimental procedures.

SUPPLEMENTAL INFORMATION

Supplemental information can be found online at https://doi.org/10.1016/j.stemcr.2023.03.013.

AUTHOR CONTRIBUTIONS

J.K.C. and B.A. wrote the manuscript with input from all the authors. J.K.C., J.W., M.V.P., and B.A. designed and interpreted the

experiments. J.K.C., L.N., Z.L., and M.T. conducted wet-lab experiments. J.K.C. and J.W. conducted dry-lab experiments. C.-C.H., M.V.P., and B.A. provided resources. B.A. was the overall supervisor.

ACKNOWLEDGMENTS

This work was supported by NIH/NIAMS grants P30AR075047 and R01AR44882 and the Irving Weinstein Foundation (to B.A); NIH/NICHD grant 1F30HD093392-01 (to J.C.); and NIH/NIGM grant T32-GM136624 (to J.W.). Analysis of cancer gene expression is based on data from the TCGA Research Network: https://www.cancer.gov/tcga.

CONFLICT OF INTERESTS

The authors have no competing interest.

Received: May 11, 2022
Revised: March 20, 2023
Accepted: March 21, 2023
Published: April 20, 2023

REFERENCES

Adam, R.C., Yang, H., Rockowitz, S., Larsen, S.B., Nikolova, M., Oristian, D.S., Polak, L., Kadaja, M., Asare, A., Zheng, D., and Fuchs, E. (2015). Pioneer factors govern super-enhancer dynamics in stem cell plasticity and lineage choice. Nature *521*, 366–370. https://doi.org/10.1038/nature14289.

Adam, R.C., Yang, H., Ge, Y., Lien, W.-H., Wang, P., Zhao, Y., Polak, L., Levorse, J., Baksh, S.C., Zheng, D., and Fuchs, E. (2018). Temporal layering of signaling effectors drives chromatin remodeling during hair follicle stem cell lineage progression. Cell Stem Cell *22*, 398–413.e7. https://doi.org/10.1016/j.stem.2017.12.004.

Basile, V., Mantovani, R., and Imbriano, C. (2006). DNA damage promotes histone deacetylase 4 nuclear localization and repression of G2/M promoters, via p53 C-terminal lysines. J. Biol. Chem. *281*, 2347–2357. https://doi.org/10.1074/jbc.M507712200.

Bonnard, C., Strobl, A.C., Shboul, M., Lee, H., Merriman, B., Nelson, S.F., Ababneh, O.H., Uz, E., Güran, T., Kayserili, H., et al. (2012). Mutations in IRX5 impair craniofacial development and germ cell migration via SDF1. Nat. Genet. *44*, 709–713. https://doi.org/10.1038/ng.2259.

Briscoe, J., Pierani, A., Jessell, T.M., and Ericson, J. (2000). A homeodomain protein code specifies progenitor cell identity and neuronal fate in the ventral neural tube. Cell *101*, 435–445. https://doi.org/10.1016/S0092-8674(00)80853-3.

Bruneau, B.G., Bao, Z.-Z., Fatkin, D., Xavier-Neto, J., Georgakopoulos, D., Maguire, C.T., Berul, C.I., Kass, D.A., Kuroski-de Bold, M.L., de Bold, A.J., et al. (2001). Cardiomyopathy in irx4-deficient mice is preceded by abnormal ventricular gene expression. Mol. Cell Biol. *21*, 1730–1736. https://doi.org/10.1128/MCB.21.5.1730-1736.2001.

Cain, C.J., Gaborit, N., Lwin, W., Barruet, E., Ho, S., Bonnard, C., Hamamy, H., Shboul, M., Reversade, B., Kayserili, H., et al. (2016). Loss of Iroquois homeobox transcription factors 3 and 5 in osteoblasts disrupts cranial mineralization. BoneKEy Rep. *5*, 86–95. https://doi.org/10.1016/j.bonr.2016.02.005.

Chen, X., Whitney, E.M., Gao, S.Y., and Yang, V.W. (2003). Transcriptional profiling of krüppel-like factor 4 reveals a function in cell cycle regulation and epithelial differentiation. J. Mol. Biol. *326*, 665–677. https://doi.org/10.1016/S0022-2836(02)01449-3.

Chen, X., Müller, G.A., Quaas, M., Fischer, M., Han, N., Stutchbury, B., Sharrocks, A.D., and Engeland, K. (2013). The forkhead transcription factor FOXM1 controls cell cycle-dependent gene expression through an atypical chromatin binding mechanism. Mol. Cell Biol. *33*, 227–236. https://doi.org/10.1128/MCB.00881-12.

Cheng, C.W., Chow, R.L., Lebel, M., Sakuma, R., Cheung, H.O.-L., Thanabalasingham, V., Zhang, X., Bruneau, B.G., Birch, D.G., Hui, C.c., et al. (2005). The Iroquois homeobox gene, Irx5, is required for retinal cone bipolar cell development. Dev. Biol. *287*, 48–60. https://doi.org/10.1016/j.ydbio.2005.08.029.

Costantini, D.L., Arruda, E.P., Agarwal, P., Kim, K.-H., Zhu, Y., Zhu, W., Lebel, M., Cheng, C.W., Park, C.Y., Pierce, S.A., et al. (2005). The homeodomain transcription factor Irx5 establishes the mouse cardiac ventricular repolarization gradient. Cell *123*, 347–358. https://doi.org/10.1016/j.cell.2005.08.004.

Gaborit, N., Sakuma, R., Wylie, J.N., Kim, K.-H., Zhang, S.-S., Hui, C.-C., and Bruneau, B.G. (2012). Cooperative and antagonistic roles for Irx3 and Irx5 in cardiac morphogenesis and postnatal physiology. Development *139*, 4007–4019. https://doi.org/10.1242/dev.081703.

Geueke, A., Mantellato, G., Kuester, F., Schettina, P., Nelles, M., Seeger, J.M., Kashkar, H., and Niemann, C. (2021). The anti-apoptotic Bcl-2 protein regulates hair follicle stem cell function. EMBO Rep. *22*, e52301. https://doi.org/10.15252/embr.202052301.

Geyfman, M., Plikus, M.V., Treffeisen, E., Andersen, B., and Paus, R. (2015). Resting no more: re-defining telogen, the maintenance stage of the hair growth cycle: resting stage of the hair follicle cycle. Biol. Rev. Camb. Phil. Soc. *90*, 1179–1196. https://doi.org/10.1111/brv.12151.

Gómez-Skarmeta, J.L., and Modolell, J. (1996). araucan and caupolican provide a link between compartment subdivisions and patterning of sensory organs and veins in the Drosophila wing. Genes Dev. *10*, 2935–2945. https://doi.org/10.1101/gad.10.22.2935.

Hopkin, A.S., Gordon, W., Klein, R.H., Espitia, F., Daily, K., Zeller, M., Baldi, P., and Andersen, B. (2012). GRHL3/GET1 and trithorax group members collaborate to activate the epidermal progenitor differentiation program. PLoS Genet. *8*, e1002829. https://doi.org/10.1371/journal.pgen.1002829.

Hsu, Y.-C., Pasolli, H.A., and Fuchs, E. (2011). Dynamics between stem cells, niche, and progeny in the hair follicle. Cell *144*, 92–105. https://doi.org/10.1016/j.cell.2010.11.049.

Hua, G., Thin, T.H., Feldman, R., Haimovitz–Friedman, A., Clevers, H., Fuks, Z., and Kolesnick, R. (2012). Crypt base columnar stem cells in small intestines of mice are radioresistant. Gastroenterology *143*, 1266–1276. https://doi.org/10.1053/j.gastro.2012.07.106.

Huang, L., Song, F., Sun, H., Zhang, L., and Huang, C. (2018). IRX5 promotes NF-κB signalling to increase proliferation, migration and invasion via OPN in tongue squamous cell carcinoma. J. Cell Mol. Med. *22*, 3899–3910. https://doi.org/10.1111/jcmm.13664.

Joost, S., Zeisel, A., Jacob, T., Sun, X., La Manno, G., Lönnerberg, P., Linnarsson, S., and Kasper, M. (2016). Single-cell transcriptomics reveals that differentiation and spatial signatures shape epidermal and hair follicle heterogeneity. Cell Syst. *3*, 221–237.e9. https://doi.org/10.1016/j.cels.2016.08.010.

Joost, S., Annusver, K., Jacob, T., Sun, X., Dalessandri, T., Sivan, U., Sequeira, I., Sandberg, R., and Kasper, M. (2020). The molecular anatomy of mouse skin during hair growth and rest. Cell Stem Cell *26*, 441–457.e7. https://doi.org/10.1016/j.stem.2020.01.012.

Kawano, M., Umeda, S., Yasuda, T., Fujita, M., Ishikawa, A., Imamura, T., Imai, T., and Nakayama, F. (2016). FGF18 signaling in the hair cycle resting phase determines radioresistance of hair follicles by arresting hair cycling. Adv. Radiat. Oncol. *1*, 170–181. https://doi.org/10.1016/j.adro.2016.05.004.

Khongkow, P., Karunarathna, U., Khongkow, M., Gong, C., Gomes, A.R., Yagüe, E., Monteiro, L.J., Kongsema, M., Zona, S., Man, E.P.S., et al. (2014). FOXM1 targets NBS1 to regulate DNA damage-induced senescence and epirubicin resistance. Oncogene *33*, 4144–4155. https://doi.org/10.1038/onc.2013.457.

Kimura-Ueki, M., Oda, Y., Oki, J., Komi-Kuramochi, A., Honda, E., Asada, M., Suzuki, M., and Imamura, T. (2012). Hair cycle resting phase is regulated by cyclic epithelial FGF18 signaling. J. Invest. Dermatol. *132*, 1338–1345. https://doi.org/10.1038/jid.2011.490.

Klein, R.H., Lin, Z., Hopkin, A.S., Gordon, W., Tsoi, L.C., Liang, Y., Gudjonsson, J.E., and Andersen, B. (2017). GRHL3 binding and enhancers rearrange as epidermal keratinocytes transition between functional states. PLoS Genet. *13*, e1006745. https://doi.org/10.1371/journal.pgen.1006745.

Leishman, E., Howard, J.M., Garcia, G.E., Miao, Q., Ku, A.T., Dekker, J.D., Tucker, H., and Nguyen, H. (2013). Foxp1 maintains hair follicle stem cell quiescence through regulation of Fgf18. Development *140*, 3809–3818. https://doi.org/10.1242/dev.097477.

Li, J., Summerlin, M., Nitiss, K.C., Nitiss, J.L., and Hanakahi, L.A. (2017). TDP1 is required for efficient non-homologous end joining in human cells. DNA Repair *60*, 40–49. https://doi.org/10.1016/j.dnarep.2017.10.003.

Liu, D., Pattabiraman, V., Bacanamwo, M., and Anderson, L.M. (2016). Iroquois homeobox transcription factor (Irx5) promotes G1/S-phase transition in vascular smooth muscle cells by CDK2-dependent activation. Am. J. Physiol. Cell Physiol. *311*, C179–C189. https://doi.org/10.1152/ajpcell.00293.2015.

Mégarbané, A., Hana, S., Mégarbané, H., Castro, C., Baulande, S., Criqui, A., Roëckel-Trevisiol, N., Dagher, C., Al-Ali, M.T., Desvignes, J.-P., et al. (2021). Clinical and molecular update on the fourth reported family with Hamamy syndrome. Mol. Syndromol. *12*, 342–350. https://doi.org/10.1159/000517253.

Nijhof, J.G.W., van Pelt, C., Mulder, A.A., Mitchell, D.L., Mullenders, L.H.F., and de Gruijl, F.R. (2007). Epidermal stem and progenitor cells in murine epidermis accumulate UV damage despite NER proficiency. Carcinogenesis *28*, 792–800. https://doi.org/10.1093/carcin/bgl213.

Rezza, A., Wang, Z., Sennett, R., Qiao, W., Wang, D., Heitman, N., Mok, K.W., Clavel, C., Yi, R., Zandstra, P., et al. (2016). Signaling networks among stem cell precursors, transit-amplifying progenitors, and their niche in developing hair follicles. Cell Rep. *14*, 3001–3018. https://doi.org/10.1016/j.celrep.2016.02.078.

Sobreira, D.R., Joslin, A.C., Zhang, Q., Williamson, I., Hansen, G.T., Farris, K.M., Sakabe, N.J., Sinnott-Armstrong, N., Bozek, G., Jensen-Cody, S.O., et al. (2021). Extensive pleiotropism and allelic heterogeneity mediate metabolic effects of IRX3 and IRX5. Science *372*, 1085–1091. https://doi.org/10.1126/science.abf1008.

Son, J.E., Dou, Z., Kim, K.-H., Wanggou, S., Cha, V.S.B., Mo, R., Zhang, X., Chen, X., Ketela, T., Li, X., et al. (2021). Irx3 and Irx5 in Ins2-Cre+ cells regulate hypothalamic postnatal neurogenesis and leptin response. Nat. Metab. *3*, 701–713. https://doi.org/10.1038/s42255-021-00382-y.

Sotiropoulou, P.A., Candi, A., Mascré, G., De Clercq, S., Youssef, K.K., Lapouge, G., Dahl, E., Semeraro, C., Denecker, G., Marine, J.-C., and Blanpain, C. (2010). Bcl-2 and accelerated DNA repair mediates resistance of hair follicle bulge stem cells to DNA-damage-induced cell death. Nat. Cell Biol. *12*, 572–582. https://doi.org/10.1038/ncb2059.

Sotiropoulou, P.A., Karambelas, A.E., Debaugnies, M., Candi, A., Bouwman, P., Moers, V., Revenco, T., Rocha, A.S., Sekiguchi, K., Jonkers, J., and Blanpain, C. (2013). BRCA1 deficiency in skin epidermis leads to selective loss of hair follicle stem cells and their progeny. Genes Dev. *27*, 39–51. https://doi.org/10.1101/gad.206573.112.

Stuart, T., Butler, A., Hoffman, P., Hafemeister, C., Papalexi, E., Mauck, W.M., Hao, Y., Stoeckius, M., Smibert, P., and Satija, R. (2019). Comprehensive integration of single-cell data. Cell *177*, 1888–1902.e21. https://doi.org/10.1016/j.cell.2019.05.031.

Sun, X., Jiang, X., Wu, J., Ma, R., Wu, Y., Cao, H., Wang, Z., Liu, S., Zhang, J., Wu, Y., et al. (2020). IRX5 prompts genomic instability in colorectal cancer cells. J. Cell. Biochem. *121*, 4680–4689. https://doi.org/10.1002/jcb.29693.

Tagami, H., Ray-Gallet, D., Almouzni, G., and Nakatani, Y. (2004). Histone H3.1 and H3.3 complexes mediate nucleosome assembly pathways dependent or independent of DNA synthesis. Cell *116*, 51–61. https://doi.org/10.1016/S0092-8674(03)01064-X.

Tang, Z., Li, C., Kang, B., Gao, G., Li, C., and Zhang, Z. (2017). GEPIA: a web server for cancer and normal gene expression profiling and interactive analyses. Nucleic Acids Res. *45*, W98–W102. https://doi.org/10.1093/nar/gkx247.

Wang, Q., Oh, J.W., Lee, H.-L., Dhar, A., Peng, T., Ramos, R., Guerrero-Juarez, C.F., Wang, X., Zhao, R., Cao, X., et al. (2017). A multi-scale model for hair follicles reveals heterogeneous domains driving rapid spatiotemporal hair growth patterning. Elife *6*, e22772. https://doi.org/10.7554/eLife.22772.

Wu, J., Lu, L.-Y., and Yu, X. (2010). The role of BRCA1 in DNA damage response. Protein Cell *1*, 117–123. https://doi.org/10.1007/s13238-010-0010-5.

Yang, H., Adam, R.C., Ge, Y., Hua, Z.L., and Fuchs, E. (2017). Epithelial-mesenchymal micro-niches govern stem cell lineage choices. Cell *169*, 483–496.e13. https://doi.org/10.1016/j.cell.2017.03.038.

Yun, J., Chae, H.-D., Choi, T.-S., Kim, E.-H., Bang, Y.-J., Chung, J., Choi, K.-S., Mantovani, R., and Shin, D.Y. (2003). Cdk2-dependent phosphorylation of the NF-Y transcription factor and its

involvement in the p53-p21 signaling pathway. J. Biol. Chem. *278*, 36966–36972. https://doi.org/10.1074/jbc.M305178200.

Zhang, D.-L., Qu, L.-W., Ma, L., Zhou, Y.-C., Wang, G.-Z., Zhao, X.-C., Zhang, C., Zhang, Y.-F., Wang, M., Zhang, M.-Y., et al. (2018). Genome-wide identification of transcription factors that are critical to non-small cell lung cancer. Cancer Lett. *434*, 132–143. https://doi.org/10.1016/j.canlet.2018.07.020.

Zhao, W., Steinfeld, J.B., Liang, F., Chen, X., Maranon, D.G., Jian Ma, C., Kwon, Y., Rao, T., Wang, W., Sheng, C., et al. (2017). BRCA1–BARD1 promotes RAD51-mediated homologous DNA pairing. Nature *550*, 360–365. https://doi.org/10.1038/nature24060.

Zhu, L., Dai, L., Yang, N., Liu, M., Ma, S., Li, C., Shen, J., Lin, T., Wang, D., Pan, W., and Li, X. (2020). Transcription factorIRX5 promotes hepatocellular carcinoma proliferation and inhibits apoptosis by regulating the p53 signalling pathway. Cell Biochem. Funct. *38*, 621–629. https://doi.org/10.1002/cbf.3517.

Zhu, Q., Pao, G.M., Huynh, A.M., Suh, H., Tonnu, N., Nederlof, P.M., Gage, F.H., and Verma, I.M. (2011). BRCA1 tumour suppression occurs via heterochromatin-mediated silencing. Nature *477*, 179–184. https://doi.org/10.1038/nature10371.

Stem Cell Reports, Volume *18*

Supplemental Information

IRX5 promotes DNA damage repair and activation of hair follicle stem cells

Jefferson K. Chen, Julie Wiedemann, Ly Nguyen, Zhongqi Lin, Mahum Tahir, Chi-Chung Hui, Maksim V. Plikus, and Bogi Andersen

Stem Cell Reports, Volume 18

Supplemental Information

IRX5 promotes DNA damage repair and activation of hair follicle stem cells

Jefferson K Chen, Julie Wiedemann, Ly Nguyen, Zhongqi Lin, Mahum Tahir, Chi-Chung Hui, Maksim V Plikus, Bogi Andersen.

Supplemental Figures

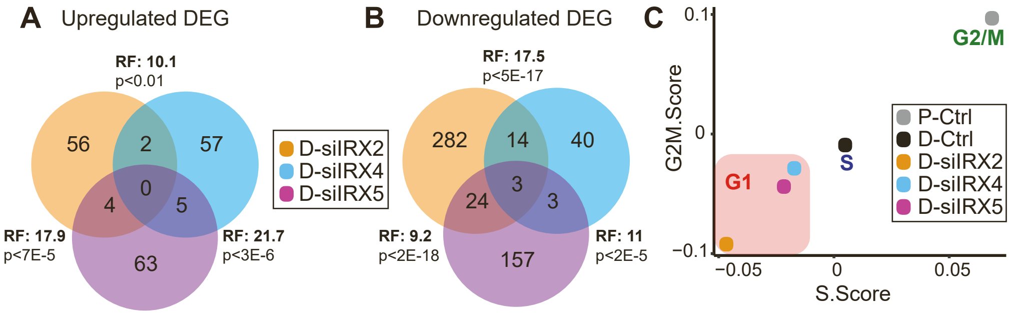
A
Upregulated DEG
RF: 10.1
p<0.01
56
2
57
0
4
5
63
RF: 17.9
p<7E-5
RF: 21.7
p<3E-6
D-siIRX2
D-siIRX4
D-siIRX5
B
Downregulated DEG
RF: 17.5
p<5E-17
282
14
40
3
24
3
157
RF: 9.2
p<2E-18
RF: 11
p<2E-5
C
G2/M
S
G1
P-Ctrl
D-Ctrl
D-siIRX2
D-siIRX4
D-siIRX5
G2M.Score
S.Score
0.1
0
−0.1
−0.05
0
0.05

Figure S1. IRX factors promote human keratinocyte proliferation, related to Figure 1. A-B) Venn diagrams showing overlap in DEG (upregulated, A; downregulated, B) after knockdowns with siIRX2, siIRX4, and siIRX5 in NHEK-D. **C**) Seurat scatter plot of cell cycle scores of control NHEK and NHEK-D after siRNA knockdowns. After knockdowns of IRX factors in NHEK-D, cell cycle gene expression becomes characteristic with the G1 stage of the cell cycle.

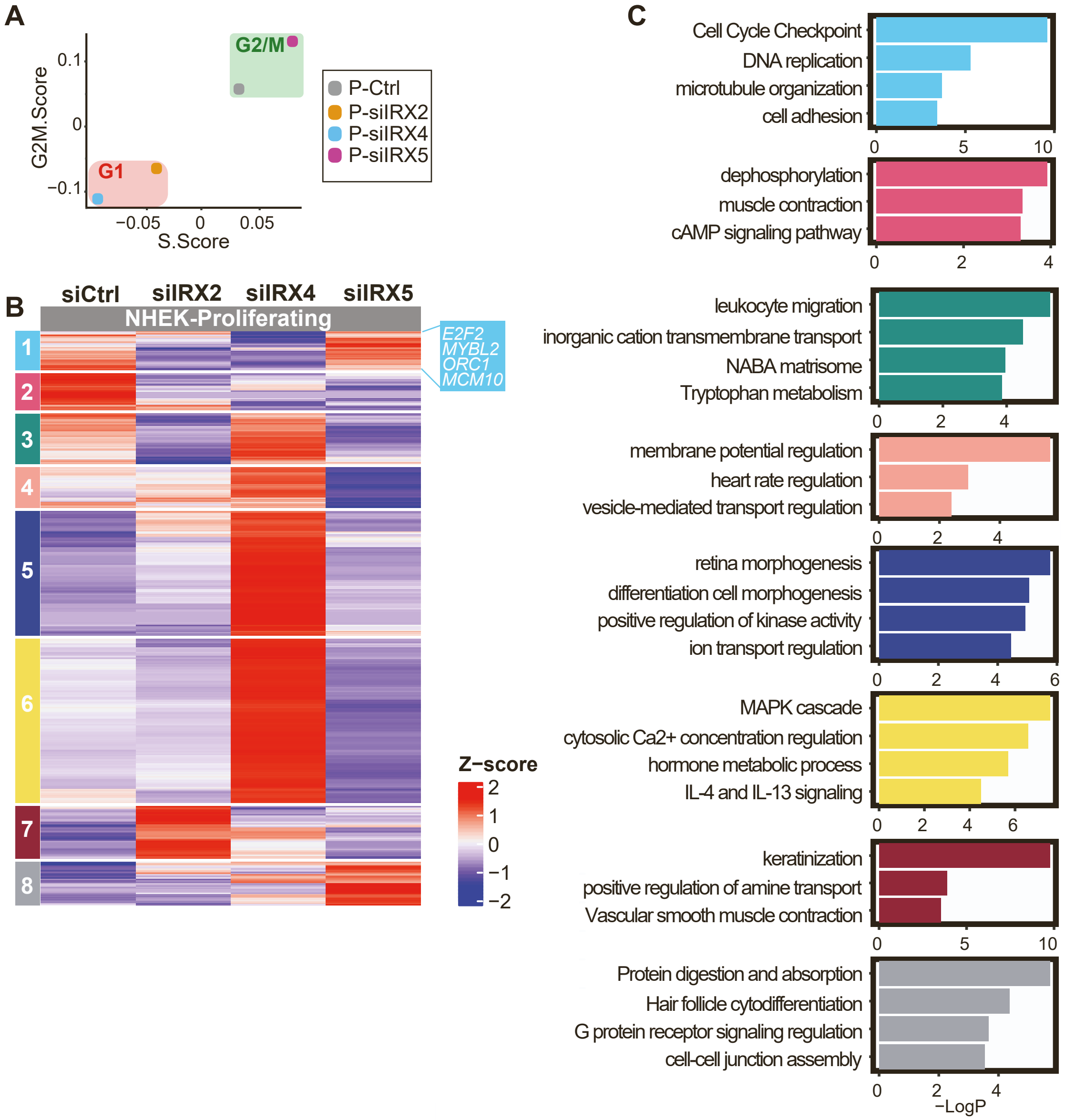

A
G2/M
G1
P-Ctrl
P-siIRX2
P-siIRX4
P-siIRX5
G2M.Score
S.Score
B
siCtrl
siIRX2
siIRX4
siIRX5
NHEK-Proliferating
E2F2
MYBL2
ORC1
MCM10
Z-score
C
Cell Cycle Checkpoint
DNA replication
microtubule organization
cell adhesion
dephosphorylation
muscle contraction
cAMP signaling pathway
leukocyte migration
inorganic cation transmembrane transport
NABA matrisome
Tryptophan metabolism
membrane potential regulation
heart rate regulation
vesicle-mediated transport regulation
retina morphogenesis
differentiation cell morphogenesis
positive regulation of kinase activity
ion transport regulation
MAPK cascade
cytosolic Ca2+ concentration regulation
hormone metabolic process
IL-4 and IL-13 signaling
keratinization
positive regulation of amine transport
Vascular smooth muscle contraction
Protein digestion and absorption
Hair follicle cytodifferentiation
G protein receptor signaling regulation
cell-cell junction assembly
−LogP

Figure S2. IRX factors promote human keratinocyte proliferation. A) Seurat scatter plot of cell cycle scores of control NHEK and NHEK-P after siRNA knockdowns. **B**) K-means clustering of the RNA-seq data in panel A. **C**) GO categories of each cluster in panel B.

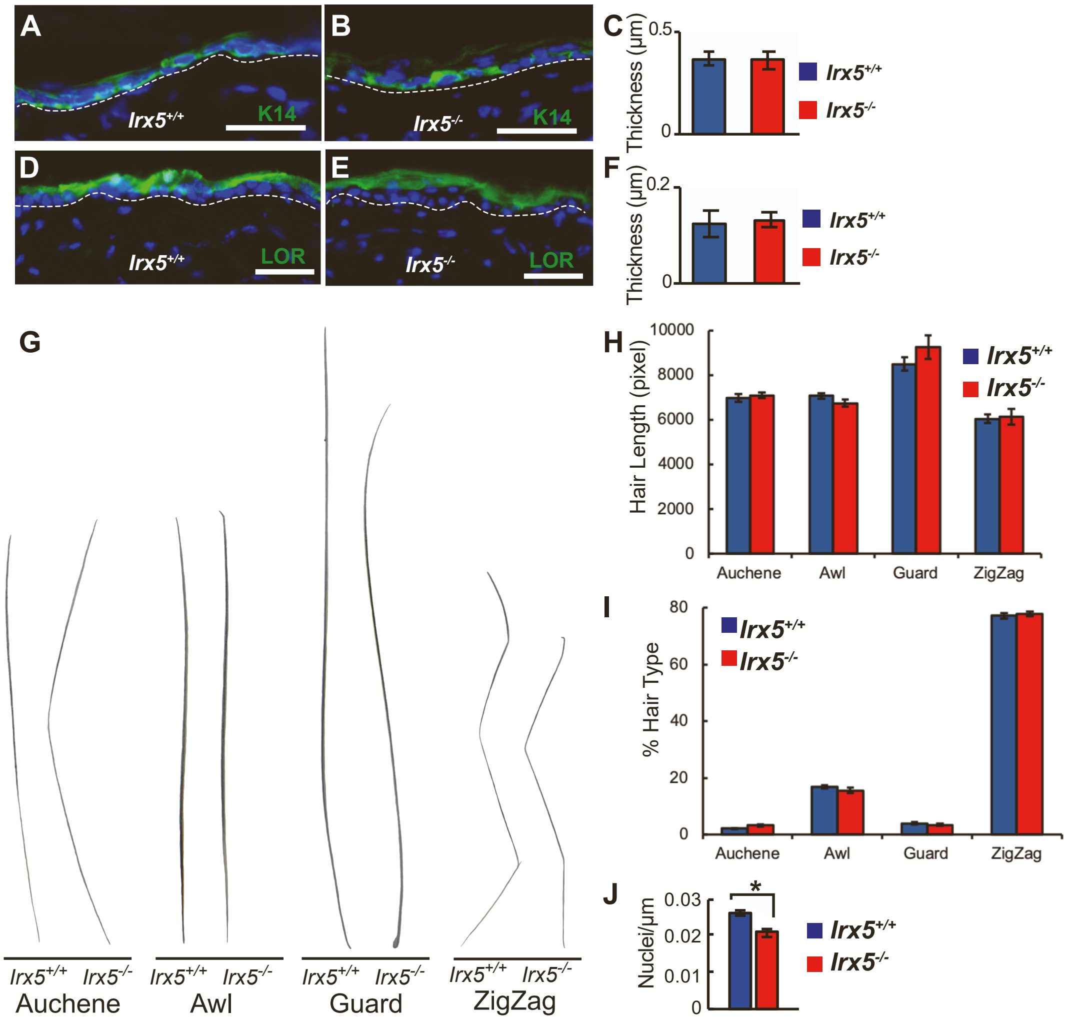
A
B
C
D
E
F
G
H
I
J
Irx5+/+
Irx5-/-
K14
LOR
Thickness (µm)
0.5
0.2
0
Hair Length (pixel)
10000
8000
6000
4000
2000
% Hair Type
80
60
40
20
Nuclei/µm
0.03
0.02
0.01
*
Auchene
Awl
Guard
ZigZag

Figure S3. Mouse epidermis and distribution of hair fiber types is normal in *Irx5*$^{-/-}$ mice, related to Figure 2. A-B) Representative images of K14 immunofluorescent staining in P20 *Irx5*$^{+/+}$ and *Irx5*$^{-/-}$ littermates. **C**) Quantification of K14 staining thickness in *Irx5*$^{-/-}$ (n=2) and *Irx5*$^{+/+}$ P20 mice (n=2). **D-E**) Representative images of LOR immunofluorescent staining in P20 *Irx5*$^{+/+}$ and *Irx5*$^{-/-}$ littermates. **F**) Quantification of LOR staining thickness, in *Irx5*$^{-/-}$ (n=2) and *Irx5*$^{+/+}$ P20 mice (n=2). RF=representative factor. **G**) Representative samples of hair fibers plucked from P20 *Irx5*$^{+/+}$ and Irx5$^{-/-}$ littermates. 100 hair fibers each from *Irx5*$^{+/+}$ (n=2) and *Irx5*$^{-/-}$ (n=2) littermates were **H**) measured to identify differences in hair length and **I**) counted to identify differences in the proportion of hair fiber types. **J**) Quantification of nuclei per 1μm length in P20 *Irx5*$^{-/-}$ (n=3) and *Irx5*$^{+/+}$ mice (n=3); Two sample t-test at 95% CI; p=0.0147.

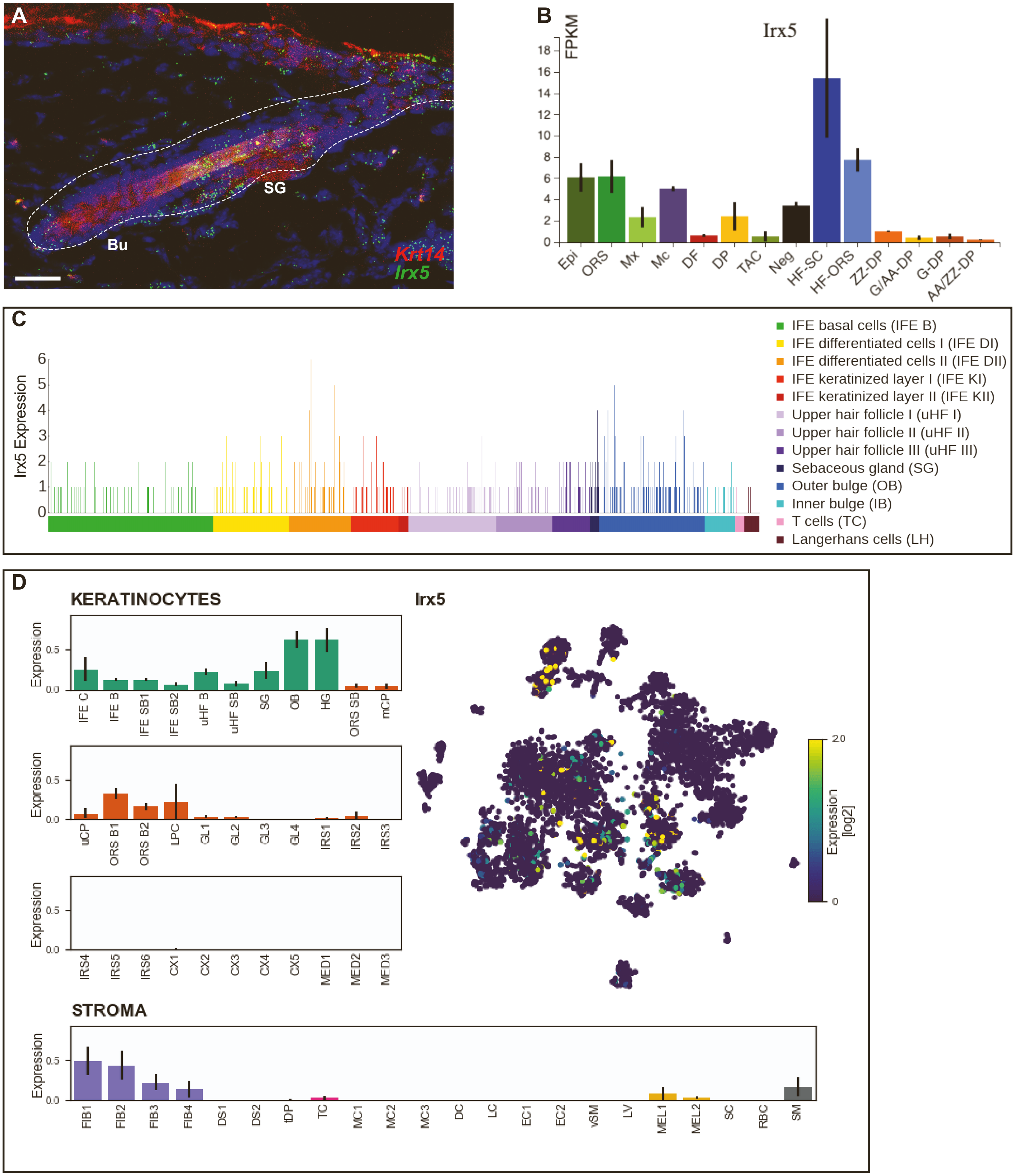
A
SG
Bu
Krt14
Irx5
B
FPKM
Irx5
Epi
ORS
Mx
Mc
DF
DP
TAC
Neg
HF-SC
HF-ORS
ZZ-DP
G/AA-DP
G-DP
AA/ZZ-DP
C
Irx5 Expression
IFE basal cells (IFE B)
IFE differentiated cells I (IFE DI)
IFE differentiated cells II (IFE DII)
IFE keratinized layer I (IFE KI)
IFE keratinized layer II (IFE KII)
Upper hair follicle I (uHF I)
Upper hair follicle II (uHF II)
Upper hair follicle III (uHF III)
Sebaceous gland (SG)
Outer bulge (OB)
Inner bulge (IB)
T cells (TC)
Langerhans cells (LH)
D
KERATINOCYTES
Irx5
Expression
IFE C
IFE B
IFE SB1
IFE SB2
uHF B
uHF SB
SG
OB
HG
ORS SB
mCP
uCP
ORS B1
ORS B2
LPC
GL1
GL2
GL3
GL4
IRS1
IRS2
IRS3
IRS4
IRS5
IRS6
CX1
CX2
CX3
CX4
CX5
MED1
MED2
MED3
Expression [log2]
STROMA
FIB1
FIB2
FIB3
FIB4
DS1
DS2
tDP
TC
MC1
MC2
MC3
DC
LC
EC1
EC2
vSM
LV
MEL1
MEL2
SC
RBC
SM

Figure S4. IRX5 is expressed in bulge cells, related to Figure 2. A) Representative sample of *Krt14* and *Irx5* RNA-FISH in normal P20 mouse hair follicle. Scale bar=20μm. Irx5 gene expression in **B**) sorted cells of P5 postnatal mouse skin from Rezza et al. (2016), **C**) second telogen mouse skin from Joost et al (2016), and **D**) mouse skin from Joost et al (2020).

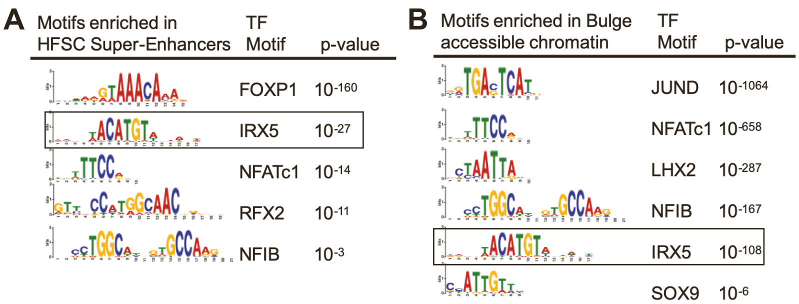
A
Motifs enriched in HFSC Super-Enhancers
TF Motif
p-value
FOXP1 10^{-160}
IRX5 10^{-27}
NFATc1 10^{-14}
RFX2 10^{-11}
NFIB 10^{-3}
B
Motifs enriched in Bulge accessible chromatin
TF Motif
p-value
JUND 10^{-1064}
NFATc1 10^{-658}
LHX2 10^{-287}
NFIB 10^{-167}
IRX5 10^{-108}
SOX9 10^{-6}

Figure S5. IRX5 motifs are enriched in HFSC gene-regulatory chromatin regions. Enriched motifs previously reported and IRX5 motif were reanalyzed using data from **A**) Adam et al. (2015) identified bulge super-enhancers and **B**) Adam et al. (2018) isolated bulge ATAC-seq.

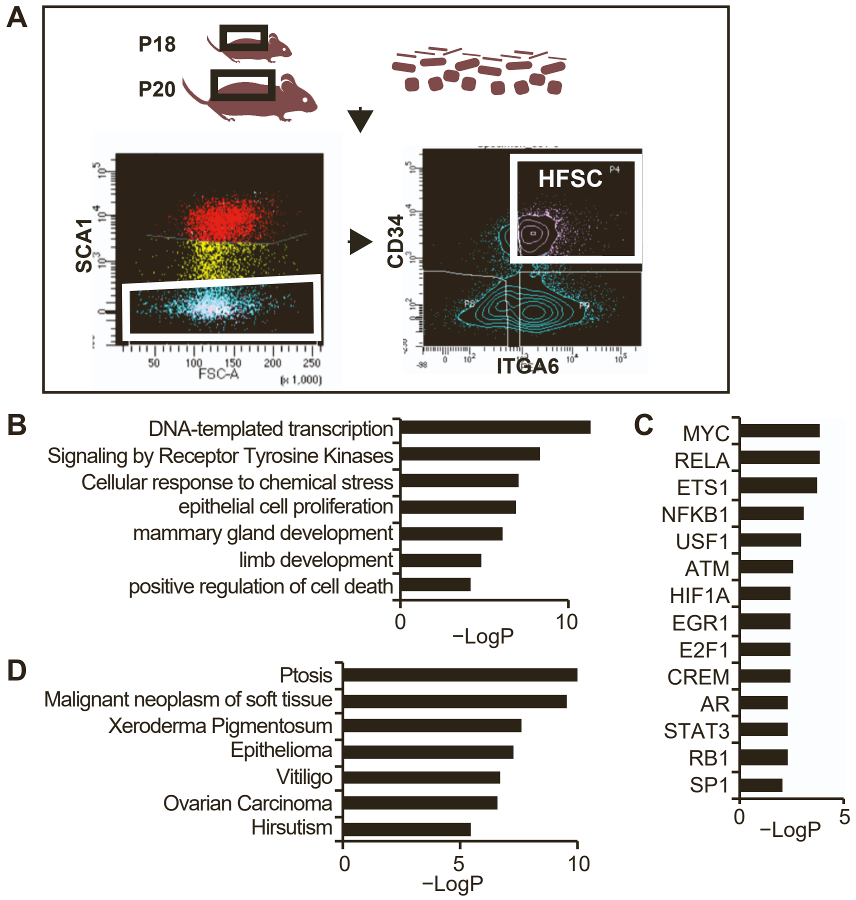

A
P18
P20
SCA1
FSC-A
CD34
HFSC
ITGA6
B
DNA-templated transcription
Signaling by Receptor Tyrosine Kinases
Cellular response to chemical stress
epithelial cell proliferation
mammary gland development
limb development
positive regulation of cell death
0
10
−LogP
C
MYC
RELA
ETS1
NFKB1
USF1
ATM
HIF1A
EGR1
E2F1
CREM
AR
STAT3
RB1
SP1
0
5
−LogP
D
Ptosis
Malignant neoplasm of soft tissue
Xeroderma Pigmentosum
Epithelioma
Vitiligo
Ovarian Carcinoma
Hirsutism
0
5
10
−LogP

Figure S6. Hair Follicle Stem Cell isolation, related to Figure 3. A) P18 and P20 *Irx5*$^{+/+}$ and *Irx5*$^{-/-}$ mice back skin was processed into a single cell suspension and FACS sorted for Sca1- CD34+ ITGA6+ HFSC. RNA sequencing was conducted with the isolated cells. *Irx5*$^{-/-}$ DEG which remained consistent between P18 and P20 (Cluster 3, 6, and 8 from Figure 3C) were analyzed together for **B**) GO, **C**) predicted transcriptional regulators, and **D**) mouse phenotype ontology.

A

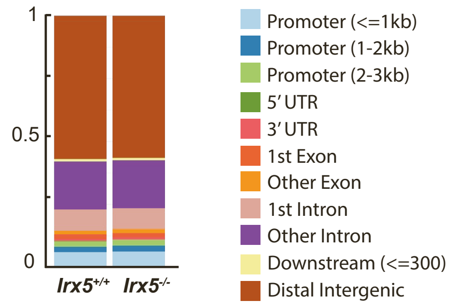

B

Motifs enriched in Irx5$^{+/+}$ HFSC open chromatin	TF Motif	p-value
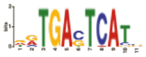	**JUND**	**10^{-415}**
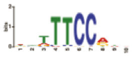	**NFATc1**	**10^{-165}**
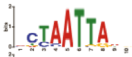	**LHX2**	**10^{-93}**
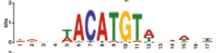	**IRX5**	**10^{-35}**
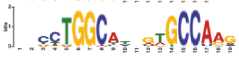	**NFIB**	**10^{-2}**
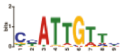	**SOX9**	**10^{-5}**

C

Motifs enriched in differential open chromatin	TF Motif	p-value
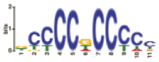	**SP1**	**10^{-397}**
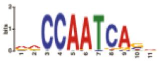	**NFYA**	**10^{-143}**
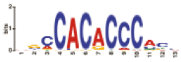	**KLF9**	**10^{-141}**
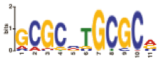	**NRF1**	**10^{-117}**
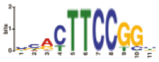	**ELK4**	**10^{-76}**
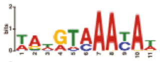	**FOXC1**	**10^{-53}**
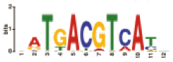	**FOSL1::JUN**	**10^{-46}**

Figure S7. P20 *Irx5*$^{-/-}$ HFSC differential open chromatin is enriched in NFY-A and SP1 binding sites, related to Figure 5. A) Genomic distribution of all open chromatin regions identified in P20 *Irx5*$^{-/-}$ HFSC and P20 *Irx5*$^{+/+}$ HFSC. **B**) Enriched motifs identified in P20 *Irx5*$^{+/+}$ HFSC. **C**) Enriched motifs identified in differential open chromatin of P20 *Irx5*$^{-/-}$ HFSC.

Supplemental Experimental Procedures

Mice

Irx5$^{-/-}$ mice and genotyping were described previously (Gaborit et al., 2012). Mice were maintained on standard 12h light cycles with ad libitum food and water. Animal experiments were performed in accordance to University of California, Irvine Institutional Animal Care and Use Committee (Protocol No. AUP-19-012). All mouse experiments were conducted on sex-matched littermates. EdU proliferation analysis was conducted with Click-iT EdU Cell Proliferation Kit (C10337). 0.5mg/mL of EdU was administered IP and mice were euthanized 2h later.

To observe anagen hair follicle growth, the back fur was shaved at P20 and mice were anesthetized and imaged every 3d. Hair follicle rescue experiments were conducted with AZD4547 (Selleckchem S2801) prepared in 4% DMSO, 5% PEG300, and 5% Tween80. AZD4547 (6mg/kg) was administered IP every 3d. Statistical analysis of back fur coverage was conducted with two-sided Kolmogorov-Smirnov test.

Tissue and Cell Isolation

P18 and P20 back skin was dissected and incubated in 0.25% trypsin (Invitrogen 1505-065) for 2h at 34C. The epidermal side was mechanically separated and resuspended in 2% FBS PBS. Debris was removed with 40uM and 70uM mesh filters. Whole epidermis samples were processed for bulk RNA-seq. For HFSC bulk RNAseq, cells were labeled with CD49f (eBioscience 12-0495), Ly-6A/E (eBioscience 45-5981-82), CD34 (eBioscience 50-0341-82), and DAPI. Ly-6A/E- CD34+ CD49f+ HFSC were isolated for bulk RNAseq and ATACseq.

Cell Culture

NHEK were obtained from donor neonatal foreskin and grown in Keratinocyte Serum Free Media supplemented with Epidermal Growth Factor and Bovine Pituitary Extract (Life Technologies). Individual Dharmacon on-TARGETplus siRNAs [negative control

(4390843), IRX2 (s45799, s45801), IRX3 (s35710, s35712), IRX4 (s55542, s27096), IRX5 (s20056, s20054)] were validated and siRNAs with at least 70% knockdown efficiency were pooled. 30nM pooled siRNAs were transfected into semi-confluent monolayers with Lipofectamine RNAi Max (Life Technologies) in OptiMEM medium. Twelve hours after transfection, 1.8mM Ca2+ was added to the medium to induce differentiation. RNA lysate was collected 72h after transfection.

Bulk RNA-sequencing

RNA quality was determined with Aglient Bioanalyzer; samples with RNA Integrity Number > 8 were used. Library preparation with Illumina TrueSeq library preparation kit and single end Illumina HiSeq 2500 sequencing was performed. Read alignment was preformed using Kallisto/0.46.2, and Deseq2 was used to identify DEG. GO was conducted using Metascape (Zhou et al., 2019).

Quantitative real time PCR was used for validation of RNA-sequencing data. cDNA was prepared with iScript cDNA kit and RT-PCR was performed with Ssofast EvaGreen (Biorad) reagent in CX384 Real-Time PCR system (Biorad). GAPDH was used to normalize gene expression between samples.

ATAC-seq

HFSC (100,000 cells/replicate) were FACS sorted and lysed according to published protocols (Buenrostro et al., 2015). Nuclei were incubated in 50uL of Tn5 transposition buffer for 30min at 37C. DNA isolation was performed with Qiagen MinElute Cleanup kit. Library preparation with Illumina TrueSeq library preparation kit and paired end Illumina HiSeq 2500 sequencing (80million reads per sample) was performed at the University of California, Irvine High Throughput Genomics Facility. FASTQC was used to check read quality. Adapters were removed with cutadapt v2.9 (parameter: -a CTGTCTCTTATACACATCT -A CTGTCTCTTATACACATCT) and sequences were aligned and mapped to mm10 (UCSC) with Bowtie2 (Langmead and Salzberg, 2012). Reads mapped to mitochondrial DNA were excluded. PCR duplicates were removed based on the list of duplicate generated from Genrich (v0.6, available at

github.com/jsh58/Genrich). Properly paired and mapped reads with mapping quality over 30 were kept for downstream analysis. To explore the feature of the uniquely mapped reads, deeptools (v3.4.2, parameter: -of bigwig --effectiveGenomeSize 2652783500 --normalizeUsing RPKM -e --ignoreDuplicates -bs 10) was used to convert aligned reads into a coverage track in BigWig format after read normalization based on RPKM. ChIPseeker (v1.24.0) was used to annotate the function of peaks and genomic region ontology was conducted with Genomic Regions Enrichment of Annotations Tool (GREAT) (McLean et al., 2010). ATACseq and RNAseq co-analysis was conducted with BETA using standard BETA plus parameters (Wang et al., 2013). Motif analysis was conducted with MEME Suit 5.5.0 using predicted IRX5 motif (JASPAR 2020 #PH0086.1).

RNA and protein detection

For immunofluorescence localization of protein, fresh tissue samples were harvested and embedded in OCT. 10uM sections were fixed in acetone at -20C for 10min, fixed in 4% PFA for 10min, permeabilized in 0.3% TritonX-100 for 10min, and blocked in 0.5% BSA PBS for 1h. Treated tissue was then incubated in primary antibody Krt14 (Abcam ab7800), Krt10 (Covance PRB-159P), and H2AX (cell signaling 9718) overnight at 4C. Secondary antibodies were incubated at room temperature for 1h. Images were captured with Keyence BZ-X700 or Nikon LSM780 confocal microscope.

For RNA FISH, fresh frozen 10uM thick OCT sections were processed and stained using RNAscope Multiplex Fluorescent Detection Kit v1 according to manufacturer's instructions. Processed samples were counterstained and preserved with ProLong Gold antifade reagent with DAPI. Images were captured on Nikon LSM780 confocal microscope.

Comparable images were post-processed in batches using the same maximum intensity projection and brightness setting for consistency with Zeiss Zen blue. Two to six biological replicates were analyzed for each marker, with each biological replicate analyzed from five to ten hair follicles. Nuclei density within the epidermis was quantified by dividing the total nuclei count by the length of the epidermis. Immunofluorescent

intensity was determined based on the RGB levels for each respective marker in the defined ROI relative to the intensity of the DAPI intensity. Each ROI was defined through the ImageJ Polygon Tool. P values were calculated using comparison of means at 95% confidence interval.

Supplemental References

Gaborit, N., Sakuma, R., Wylie, J.N., Kim, K.-H., Zhang, S.-S., Hui, C.-C., and Bruneau, B.G. (2012). Cooperative and antagonistic roles for Irx3 and Irx5 in cardiac morphogenesis and postnatal physiology. Development *139*, 4007-4019. 10.1242/dev.081703.

Langmead, B., and Salzberg, S.L. (2012). Fast gapped-read alignment with Bowtie 2. Nature Methods *9*, 357-359. 10.1038/nmeth.1923.

McLean, C.Y., Bristor, D., Hiller, M., Clarke, S.L., Schaar, B.T., Lowe, C.B., Wenger, A.M., and Bejerano, G. (2010). GREAT improves functional interpretation of cis-regulatory regions. Nature Biotechnology *28*, 495-501. 10.1038/nbt.1630.

Wang, S., Sun, H., Ma, J., Zang, C., Wang, C., Wang, J., Tang, Q., Meyer, C.A., Zhang, Y., and Liu, X.S. (2013). Target analysis by integration of transcriptome and ChIP-seq data with BETA. Nature Protocols *8*, 2502-2515. 10.1038/nprot.2013.150.

Zhou, Y., Zhou, B., Pache, L., Chang, M., Khodabakhshi, A.H., Tanaseichuk, O., Benner, C., and Chanda, S.K. (2019). Metascape provides a biologist-oriented resource for the analysis of systems-level datasets. Nature Communications *10*, 1523. 10.1038/s41467-019-09234-6.
